# Activable Multi-Modal Nanoprobes for Imaging Diagnosis and Therapy of Tumors

**DOI:** 10.3389/fchem.2020.572471

**Published:** 2021-04-12

**Authors:** Yan Yang, Saisai Yue, Yuanyuan Qiao, Peisen Zhang, Ni Jiang, Zhenbo Ning, Chunyan Liu, Yi Hou

**Affiliations:** ^1^ College of Life Science and Technology, Beijing University of Chemical Technology, Beijing, China; ^2^ Key Laboratory of Colloid, Interface and Chemical Thermodynamics, Institute of Chemistry, Chinese Academy of Sciences, Beijing, China; ^3^ School of Chemistry and Chemical Engineering, University of Chinese Academy of Sciences, Beijing, China; ^4^ Institute of Atmospheric Physics, Chinese Academy of Sciences, Beijing, China

**Keywords:** tumor theranostics, microenvironment, target-triggering, multi-modal probe, nanomedicine

## Abstract

Malignant tumors have become one of the major causes of human death, but there remains a lack of effective methods for tiny tumor diagnosis, metastasis warning, clinical efficacy prediction, and effective treatment. In this context, localizing tiny tumors via imaging and non-invasively extracting molecular information related to tumor proliferation, invasion, metastasis, and drug resistance from the tumor microenvironment have become the most fundamental tasks faced by cancer researchers. Tumor-associated microenvironmental physiological parameters, such as hypoxia, acidic extracellular pH, protease, reducing conditions, and so forth, have much to do with prognostic indicators for cancer progression, and impact therapeutic administrations. By combining with various novel nanoparticle-based activatable probes, molecular imaging technologies can provide a feasible approach to visualize tumor-associated microenvironment parameters noninvasively and realize accurate treatment of tumors. This review focuses on the recent achievements in the design of “smart” nanomedicine responding to the tumor microenvironment-related features and highlights state-of- the-art technology in tumor imaging diagnosis and therapy.

## Introduction

Effective precision cancer diagnosis and therapy remain as critical challenges in current tumor studies and clinics. The prognostic factors of malignant tumors, including proliferation, invasion, and metastasis, are closely associated with variations in physiological parameters, such as hypoxia, low extracellular pH, enzymes, and reducing conditions (Ma et al., 2018b). For instance, the lowered extracellular pH induced by the enhanced glucose uptake and altered glucose metabolism of tumor cells is deemed to be a hallmark of cancer, because it can promote angiogenesis in tumor tissue, and accelerate the degradation of the extratumoral matrix by affecting proteolytic enzymes (Ma et al., 2018a). Thus, monitoring the parameters and clarifying their relationship is critical not only for tumor diagnostics, but also for therapeutic administrations.

The current characterizations of tumor-associated microenvironments mostly rely on *in vitro* analysis of the secreted proteins and gene expression, or invasive methods such as microelectrodes (Anh-Nguyen et al., 2016; Zhang et al., 2018; Song Y. et al., 2019). Although these methods can provide accurate molecular information, they are not able to provide complete information on the tumor microenvironment due to its complexity and spatiotemporal heterogeneity. In this context, tumor microenvironment analysis still needs advanced diagnostic techniques to offer exhaustive information on the tumor progression.

The development of noninvasive molecular imaging techniques and probes provides new insights into this area by providing versatile engineered molecular imaging probes for noninvasively monitoring the tumor microenvironment (Mi et al., 2016; Zhang P. et al., 2019b; Wang et al., 2020). In principle, molecular imaging can adopt different imaging techniques such as computed tomography (CT), magnetic resonance imaging (MRI), nuclear imaging, optical imaging, and so forth. The molecular imaging based on recognition between the exogenous probes and molecular marks associated with tumors can provide spatiotemporal information of the tumor at a cellular or even molecular level (Weissleder, 1999; Weissleder and Ntziachristos, 2003). The tumor microenvironment physiological parameters are important hallmarks, thus rendering them attractive targets to design target-triggering probes that can respond to stimuli in the tumor microenvironment based on novel chemical designing (Mura et al., 2013; Gao et al., 2017; Ma et al., 2018b).

A series of molecular imaging probes, especially nanoparticle (NP)-based probes, have been emerging rapidly as potential precision tumor theranostic agents, as they are able to not only serve as imaging agents to improve the diagnosis’ accuracy, but also provide a platform for innovative tumor therapies (Janib et al., 2010; Kelkar and Reineke, 2011; Omidi, 2011). Regarding the therapeutics of tumors, current clinical cancer therapies mainly rely on surgical resection, chemotherapy, and radiotherapy. However, it remains a challenge to remove tumors precisely and completely through oncological surgery, due to the extreme difficulty in identifying the tumor boundary (Chen Q. et al., 2019), while chemo drugs and radiation would lead to serious side effects (Oun et al., 2018). In order to improve therapeutic efficacy and minimize the side-effects, several innovative nano-based tumor therapies are being developed, including targeted chemotherapy with controlled release of anti-cancer agents, chemodynamic therapy (CDT), nanovaccine-based immunotherapy, gene therapy mediated by nanocarriers, and nano-enhanced physical therapy such as photothermal therapy (PTT), photodynamic therapy (PDT), magnetic hyperthermia (MHT), and radiotherapy.

This review focuses on the state-of-the art of target-triggered nanoprobes for tumor theranostics; we will summarize the preparation strategies, response mechanisms, and theranostic applications of state-of-the art activable theranostic nanoprobes.

## *Cancer* Theranostic Probes for Controlled Release of Chemical Anticancer Agents

To endow the tumor imaging nanoprobes with the abilities of loading and controlling the release of anticancer chemicals is one of the most basic and widely used approaches for designing cancer theranostics nanoprobes (Chen et al., 2018; Gu et al., 2019). On the one hand, nanomaterials can be employed as excellent signal carriers due to their various intrinsic optical and magnetic properties to sensitively detect the tumors (Jing et al., 2020). One the other hand, nano-delivery systems can overcome the limitations of rapid nonspecific clearance and poor biodistribution of conventional low-molecular-weight antitumor drugs by packaging the chemical agents within sterically stabilized, long-circulating nanoformulations, which can be further surface-modified with ligands to actively target cellular/molecular components of the tumors (Sumer and Gao, 2008; Saha, 2009; Tee et al., 2019).

### Using Nanocarriers to Improve Conventional Chemotherapy Drugs for Tumor Theranostics

As is well known, the clinical application of chemotherapy is impeded by its unsatisfactory efficacy and severe side effects (Rezaee et al., 2017; Peters, 1994). In this regard, several cancer nanomedicines have been developed through loading the chemotherapeutics on nanoagents aimed at reducing side effects, and are currently used in clinic (Bobo et al., 2016). For example, the FDA has approved anticancer nanodrugs including Doxil (PEGylated liposomal encapsulation of the anticancer drug doxorubicin) for ovarian cancer treatment (1995) and Abraxane (albumin-bounded paclitaxel nanoparticles) for breast cancer treatment (2005), metastatic non-small-cell lung cancer (2012), and metastatic pancreatic cancer (2013), which significantly alleviate the adverse effects of patients in clinical trials (Barenholz, 2012). However, even though the delivery of cytotoxic chemotherapeutics through nanocarriers can enhance drug tolerability in patients as compared to the conventional formulations, the survival benefit of them are still modest (Petersen et al., 2016). In addition, long-term side effects associated with nanomedicines should be considered (Schottler et al., 2016). A large proportion of intravenously administered nanoparticles will be finally captured by the immune system of an organism and then retained in the reticuloendothelial system (RES, e.g., liver and spleen), which could cause potential risks of toxicity due to long-term retention if they are difficult to metabolize, largely hampering their practical application (De Jong et al., 2008; Poon et al., 2019).

**FIGURE 1:**
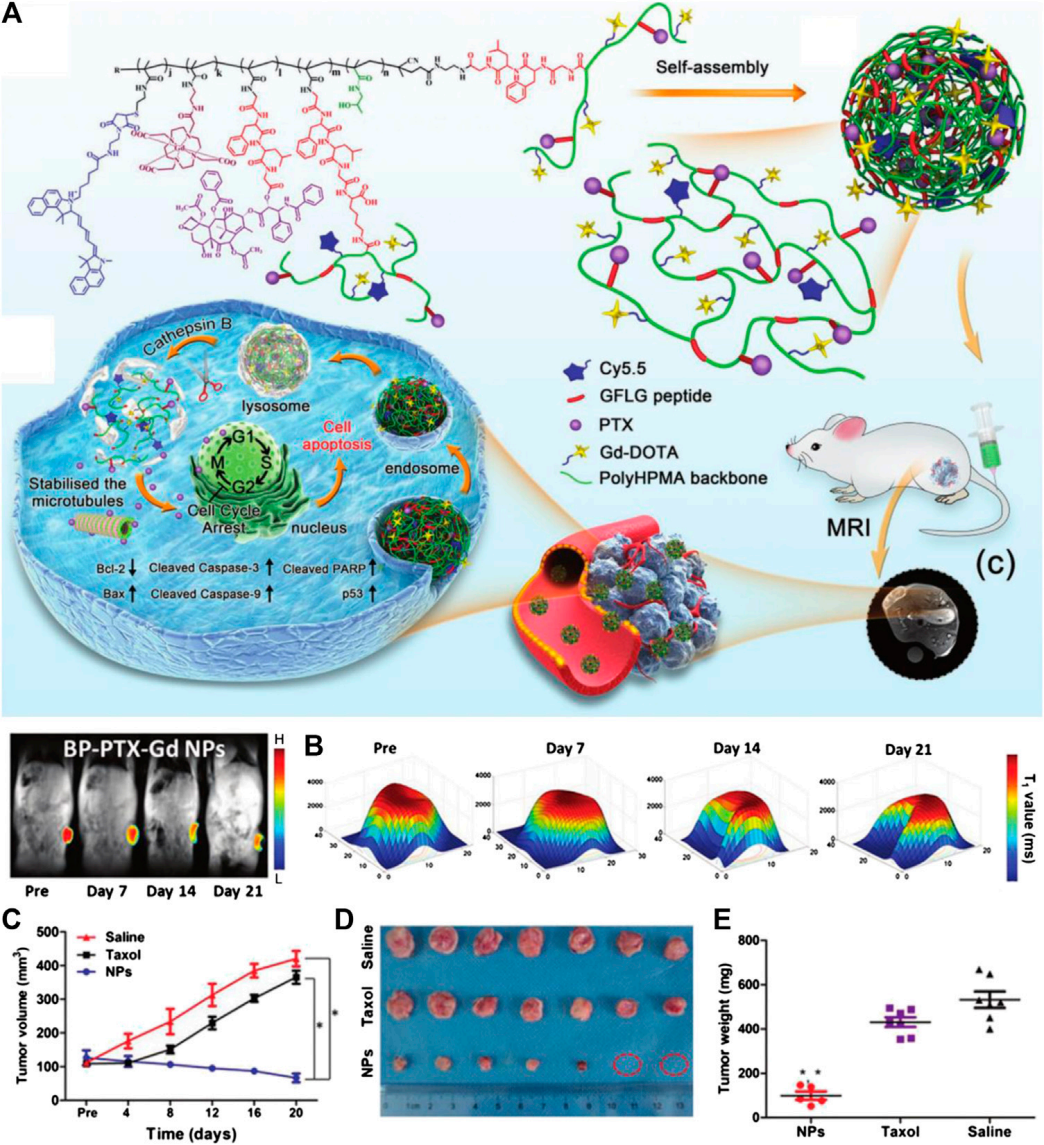
**(A)** Schematic illustration of cathepsin B-responsive biodegradable theranostic nanomedicine derived from branched pHPMA. **(B)** Distribution of *T*_1_ value of tumor at different time points after injection. **(C)** Quantitative tumor volume changes of mice obtained by MRI after being treated with saline, Taxol, and BP-PTX-Gd NPs (*n* = 7, *p < 0.001 vs. saline, *p < 0.001 vs. Taxol). **(D)** Images of the tumors harvested from the mice 21 days after the treatment. **(E)** The tumor weight of tumor-bearing mice after treatment (*p < 0.001 vs. saline, *p < 0.001 vs. Taxol). Reproduced with permission from Cai H. et al. (2020). Copyright 2020 Wiley-VCH Verlag GmbH & Co. KgaA.

To overcome these limitations, nano-systems incorporating stimuli-responsive materials have been designed to selectively release anticancer agents within tumor regions, and can keep silent in normal tissue to greatly avoid off-target side effects. In addition, due to the properties of nanocarriers, these controlled drug release systems can be further combined with tumor imaging to accurately track the drug delivery routes. Based on this concept, fabricating the imaging moiety and the anticancer prodrug together is the most commonly used design strategy for these probes. For example, Luo and coworkers prepared a stimuli-responsive polymeric nano-prodrug for tumor theranostics through imaging moieties (gadolinium-chelates) and therapeutic moieties (PTX). Such branched polymeric PTX-Gd-based nanoparticles (BP-PTX-Gd NPs) exhibit good biocompatibility and high stability under physiological conditions, but can be triggered to degrade in the tumor microenvironment and release PTX. On an animal level, BP-PTX-Gd NPs can not only significantly improve the MRI contrast of tumors due to the high *r*_1_ value (8.6 mM^−1^ s^−1^), but also possess a satisfying therapeutic effect on tumors, which is much better than that of Taxol, a clinical anticancer drug (Figure 1) (Cai H. et al., 2020). In another work, Song and coworkers reported a sequential pH and reduction-responsive hybrid assembly composed of polymer and gold nanorod (AuNR) to overcome the biological barriers via a two-stage size decrease and disassembly through responding to the specified tumor microenvironment. After tumor uptake, the ultrasmall AuNRs with polymerized reduction-responsive DOX prodrug on their surface will disassemble, and then penetrate into the deep area of solid tumors and realize the release of DOX. In the meantime, these hybrid nanoparticles can be employed as satisfactory deep-tissue PA and surface-enhanced Raman scattering imaging agents for real-time monitoring of physiological behaviors of tumors during nano-based treatments (Liu T. et al., 2019).

Another interesting strategy is to make the imaging signal also capable of responding to stimuli (Han et al., 2020). As the probe reaches the tumor regions, the prodrug and the imaging module can be simultaneously activated, thereby realizing the specific diagnosis and treatment of the tumor (Mura et al., 2013; Lu et al., 2016). Through this approach, the probes can not only realize the theranostics of tumor, but also readily monitor drug release by the activated imaging signal enhancement in a spatiotemporally concurrent manner, which makes imaging the activity and intratumoral distribution of drugs easier, thus enhancing the therapeutic efficacy and prognostic evaluation. Huang and coworkers report a manganese-iron layered double hydroxide (MnFe-LDH) to serve as a pH-responsive nanoplatform for cancer theranostics. Such a platelet-like nanoplatform can be successfully triggered by the lower pH in the microenvironment of a solid tumor to release paramagnetic Mn^2+^ and Fe^3+^ ions, thereby enhancing the *T*_1_ MR signal of *T*_1_ of tumor sites. More importantly, the layered structure endows MnFe-LDH with pH-controlled chemotherapeutic drug methotrexate (MTX) loading capacity, which can effectively kill the tumor cells (Huang et al., 2017). Similarly, manganese dioxide (MnO_2_) nanoparticles were prepared and stabilized by bovine serum albumin (BSA), which was further coated with a nanoscale coordination polymers (NCP)-shell composed of a high atomic number of Hf ions and cisplatin prodrug c,c,t- (diamminedichlorodisuccinato)Pt (IV) (DSP). After further protection from polyethylene glycol (PEG), the formed BM@NCP(DSP)-PEG can not only diagnose the tumor through MRI by Mn^2+^ release in an acidic tumor microenvironment, but also serve as a radio-sensitizer due to the strong X-ray attenuation ability of Hf to realize radiotherapy, together with a chemotherapeutic drug attributed to the release of cisplatin. Meanwhile, the excess O_2_ will be generated by the tumor endogenous H_2_O_2_ *in situ* under the MnO_2_ catalysis, which can overcome the radio-resistance caused by hypoxia to enhance the efficacy (Liu et al., 2017). In another work, Chen and coworkers reported a kind of GSH-responsive nanovesicles with a yolk-shell structure, in which both therapeutic drugs (DOX) and MRI contrast agents were both encapsulated inside the nanovesicles. The obtained nanovesicles with restrained drug activity and quenched *T*_1_ MRI contrast ability can respond to GSH in a tumor microenvironment and lead to both *T*_1_ contrast activation and DOX release, thereby monitoring drug release by activated *T*_1_ MRI signal (Liu D. et al., 2020).

### Tumor Probe Integrated With Imaging and Chemodynamic Therapy

The Fenton reaction, which can be simply defined as the formation of hydroxide (OH^−^) and highly oxidative hydroxyl (•OH) radical by a reaction between ferrous ion (Fe^2+^) and hydrogen peroxide (H_2_O_2_), has been widely used to degrade the organic pollutant (Gupta et al., 2016).

$${\text{Fe}}^{2+}+{\text{H}}_{2}{\text{O}}_{2}\to {\text{Fe}}^{3+}+{\text{OH}}^{-}+•\text{OH}$$

Apart from the catalytic effect of Fe ions, other transition metal ions, such as Mn^2+^, Ti^3+^, Cu^2+^, or Co^2+^ ions, can also be employed as catalytic ions of the reaction to generate •OH from H_2_O_2_, which is known as the Fenton-like reaction. Based on this mechanism, chemodynamic therapy (CDT), a kind of *in situ* tumor treatment approach, is proposed to kill cancer cells through excessive •OH produced by Fenton or Fenton-like reaction in tumor sites (Tang et al., 2018). Specifically, transition metal ions contain nanomaterials that can be designed to release these ions under the specific tumor microenvironment and initiate the Fenton or Fenton-like reaction to decompose the tumor endogenous H_2_O_2_, thus accumulating the •OH to trigger apoptosis of tumor cells (Zhang et al., 2016). Notably, Fenton or Fenton-like reaction will be significantly inhibited in the weak alkaline physiological conditions or in the normal tissues with insufficient H_2_O_2_. Therefore, compared with general radiotherapy and chemotherapy, CDT shows better tumor selectivity and thus avoids off-target side effects.

Based on this tumor therapy, one feasible strategy to design theranostic nanoprobes is to combine CDT with MRI. The paramagnetic ions, such as Mn^2+^, Fe^3+^, or Cu^2+^, are not only the reactants or products of Fenton or Fenton-like reactions, but also excellent *T*_1_-MRI contrast agents owing to their long electron spin relaxation times and high magnetic moments, providing a natural bridge between tumor diagnosis and treatment.

**FIGURE 2:**
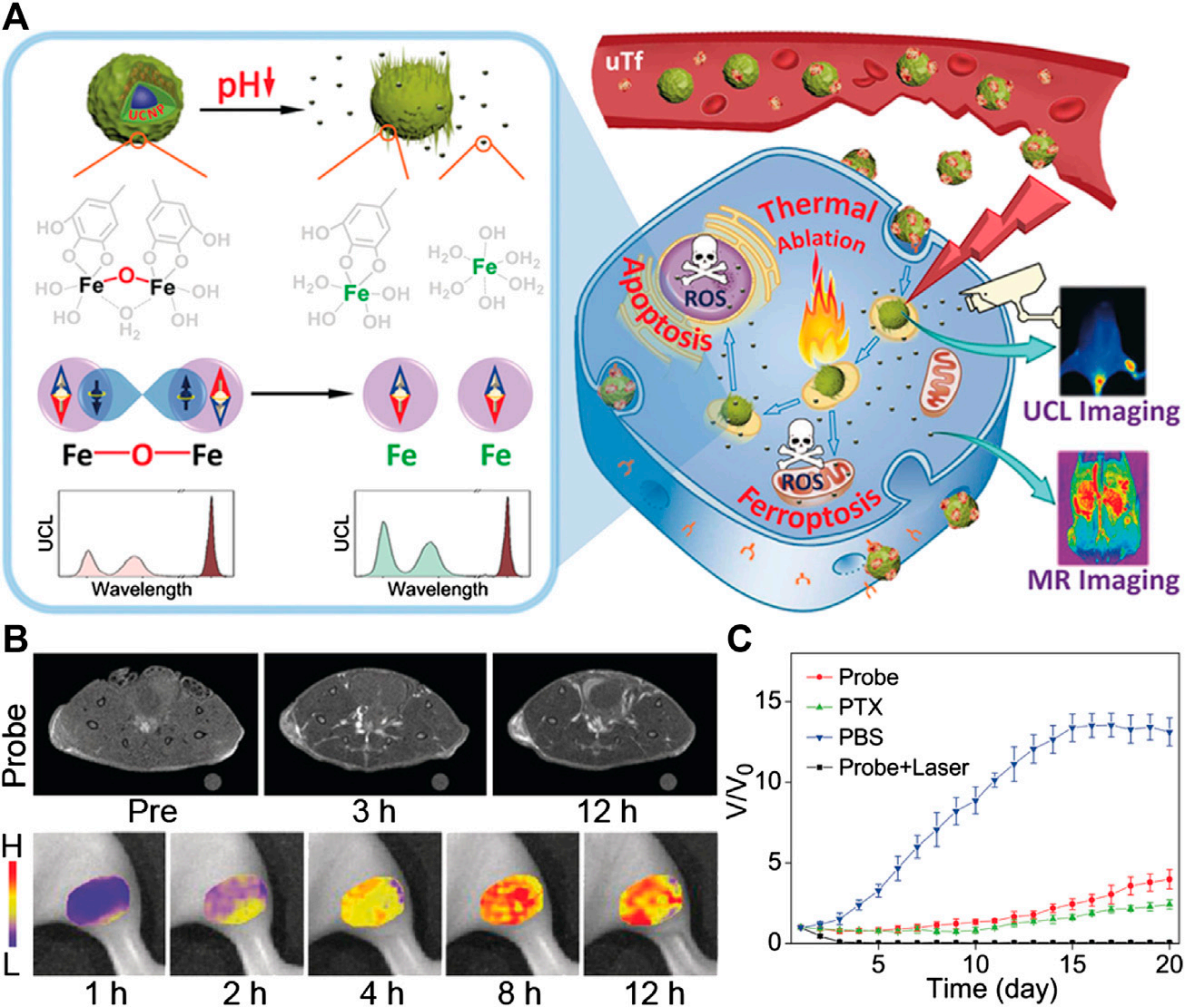
**(A)** Illustration to demonstrate the activatable function of UCNP@GA-FeIII probe for MRI and its therapeutic function involving multiple pathways. **(B)** *T*_1_-weighted MR images of tumor-bearing mice acquired at different time points pre- and post-injection of UCNP@GA-FeIII probe **(upper)**, and mapping of Fe^3+^ release based on I_475_/I_800_ ratio of upconversion luminescence **(bottom)**. **(C)** the tumor growth curves in different groups of mice after treatment. Reproduced with permission from Zhang P. et al. (2019a). Copyright 2019 Wiley-VCH Verlag GmbH & Co. KgaA.

Iron ion is the first reported ion that can trigger the Fenton reaction within a tumor area. Due to the existence of various redox molecules, such as ROS an GSH, in the tumor microenvironment, Fe^3+^ and Fe^2+^ can readily transform to each other, thus leading to a stronger catalytic effect on H_2_O_2_, and causing a large accumulation of ROS in cancer cells, which is also known as Ferroptosis (Dixon et al., 2012; Schoenfeld et al., 2017; Zheng et al., 2017). However, as a kind of paramagnetic ion, Fe^3+^ can significantly reduce the relaxation time of 1H protons of surrounding water molecules, thereby selectively lightening the tumor in *T*_1_-weighted MR images. Accordingly, several Fe-based nanoprobes, which are able to release Fe^3+^ in the tumor area, have been widely studied as Fenton reagents and tumor CDT initiators (Huo et al., 2019; Zhao P. et al., 2019; Du et al., 2020). However, the harsh reaction requirement of a low pH (∼3–4) and the slow reaction rate (63 m^−1^ s^−1^) still limits their practical application in tumor theranostics. To overcome this problem, Bu and coworkers successfully accelerated the intratumoral Fenton process with the help of a temperature increase generated by photothermal treatment to improve the therapeutic effect of CDT. They prepared an antiferromagnetic pyrite polyethylene glycol (FeS_2_-PEG) nanocube, which was activated by peroxide, and formed a valence-variable elemental Fe layer on the surface through self-oxidation, inducing both the self-enhanced MRI and photothermal enhanced CDT. Such a designed FeS_2_-PEG can catalyze H_2_O_2_ disproportionately to efficiently generate •OH, leading to a specific CDT. The Fe^3+^ on the surface layer of the nanocube after self-oxidation can enhance the *T*_1_ and *T*_2_ relaxation and reports the H_2_O_2_ content in tumor through MR signal enhancement. (Tang et al., 2017). On the basis of this study, Gao and coworkers proposed another approach to further improve the CDT efficacy under the premise of heating. They found that the coordinatively unsaturated complex formed by gallic acid and Fe^3+^ will become less stable in an acidic pH range, and can be readily triggered by a weak acid microenvironment to slowly decompose, thus releasing Fe^3+^. Based on this approach, they designed coordinatively unsaturated Fe(III) complex-based activatable nanoprobes toward activable tumor MR imaging and therapy (Figure 2). Through coating upconversion luminescence (UCL) nanoparticles with the coordinatively unsaturated GA-Fe(III) complex, the release of Fe^3+^ can be triggered by the low pH of the microenvironment and can be monitored by UCL at different wavelengths. Through the comparison of optical and MR imaging of the tumor area, it was confirmed that the release of Fe^3+^ within the tumor area can significantly boost the *T*_1_ contrast of tumors. Apart from this activable MRI capacity, the released Fe^3+^ in tumor can also generate free radicals in tumor cells through catalyzing the Fenton reaction, while the remaining GA-Fe(III) complex on the surface of the nanoprobe can still realize the photothermal conversion for PTT to further enhance the therapeutic effect of the tumor. This work thus provides a novel design integrating the activable MRI ability and tumor treatment with multiple pathways of Fe-based PTT/CDT (Zhang P. et al., 2019a).

Similar to Fe^3+^, a number of other ions-based nanomaterials have been developed to serve as Fenton reagents and MRI contrast agents, which also exhibited great potential for CDT because of their ROS generation and *T*_1_-weighted MRI signal enhanced abilities in tumor sites because of ion release. For example, Cu (I)-based nanomaterials are more adaptable to the slightly acidic tumor microenvironment with high •OH production. The reaction rate of the Cu-induced Fenton-like reaction is much greater (1 × 10^4^ M^−1^ s^−1^) than that of a Fe-based Fenton reaction in the microenvironment (Cai et al., 2019; Hu R. et al., 2019). Liu and coworkers fabricated a highly efficient Cu-based Fenton-like reagent Cu_3_P nanocrystal for tumor theranostics. Such nanocrystals could respond to over-expressed H_2_O_2_ in tumor sites and generate a large amount of toxic •OH through Fenton-like reaction through Cu (I) catalysis. The Cu_3_P nanocrystal also possess considerable photothermal conversion effects in the NIR II region, which can not only serve as a PAI agent to detect the tumor, but also significantly improve the efficiency of Fenton-like reaction, achieving good synergistic therapeutic effects of PTT and CDT. In addition, the product of the Fenton-like reaction, paramagnetic Cu(II), can be employed as a satisfactory *T*_1_ contrast agent for *in situ* self-generation MRI (Liu Y. et al., 2019b).

More recently, Li and coworkers reported a biomimetic CS-GOD@CM nanocatalyst for breast cancer CDT. Such nanomaterials, which contain Cu_2−x_Se nanoparticles, glucose oxidase, and tumor cell membrane, can effectively improve the Fenton reaction through increasing the H_2_O_2_ concentration within tumors. With the monitoring of PAI, as the amount of H_2_O_2_ in the tumor reaches its peak value, the second near-infrared (NIR-II) light irradiation was employed to further boost the efficacy of tumor treatment at the maximum concentration, which is monitored by photoacoustic imaging. The magnified Fenton-like reaction can produce a large amount of radicals and exhibits an excellent therapeutic efficacy of breast cancer (Wang T. et al., 2019).

As mentioned before, although the tumor microenvironment exhibits the characteristics of a low pH and H_2_O_2_ overproduction, it also has a higher concentration of GSH (up to 10 × 10^−3^ M) with •OH scavenging ability, which largely limits the CDT effect. To address this problem, Chen and coworkers developed self-reinforcing CDT nano-agents MS@MnO_2_ through encapsulating mesoporous SiO_2_ with MnO_2_, which possesses both Mn^2+^ release and GSH consumption properties. Such nano-agents can be employed to design a tumor-microenvironment- triggered theranostic platform for MRI-monitored cancer therapy. In the presence of HCO^3-^ in physiological conditions, Mn^2+^ can accelerate Fenton-like reaction to generate •OH from endogenous H_2_O_2_ in a tumor microenvironment. The GSH depletion properties of MnO_2_ renders cancer cells more vulnerable to •OH, which further ensures the effective CDT of MS@MnO_2_ NPs. On the other hand, MS@MnO_2_ NPs can also be used as activable MRI contrast agents owing to the GSH-triggered generation of paramagnetic Mn^2+^ *T*_1_, which is suitable for monitoring the CDT process of a tumor (Lin et al., 2018).

To further enhance the therapeutic effect on tumors, CDT can be combined with chemotherapy, as discussed in the previous section, to fight against tumors through both reactive oxygen species (ROS) and chemotherapeutic drugs (Liu et al., 2019). Chen and coworkers developed a nanomedicine (LaCIONPs) for cancer chemo/chemodynamic combination therapy, which can realize microenvironment-triggered tumor-specific chemotherapeutic drug release and ROS production. The LaCIONPs are prepared through assembling and encapsulating the iron oxide nanoparticles (IONPs) and lapachone (La) in a nanostructure via H_2_O_2_-responsive polyprodrug and pH-responsive polymer, which can be further labeled by radionuclide to identify the tumor and trace the biodistribution of nanoagents *in vivo* through PET imaging. Once the LaCIONPs are ingested by tumor cells, their structure will be decomposed in acidic lysosomes, leading to the rapid release of both La and iron ions, in which La can generate H_2_O_2_ through tumor specific catalysis to react with Fe ions and generate highly toxic •OH for CDT. In addition, H_2_O_2_ also activates the release of chemotherapeutic drug camptothecin for chemotherapy (Wang S. et al., 2019). In another work, an organic theranostic nanomedicine (PTCG NPs) is constructed through the coordination chemistry between Fe^3+^ ion and epigallocatechin-3-gallate (EGCG), phenolic platinum (IV) prodrug (Pt-OH), and polyphenol modified block copolymer (PEG-b-PPOH). Such PTCG NPs possess high stability in the blood stream due to their stable metal-polyphenol coordination, but can efficiently release cisplatin after ingestion by tumor cells. Meanwhile, the released Fe^3+^ can catalyze H_2_O_2_ to produce free radicals to kill cancer cells. Interestingly, apart from the chemotherapeutic effect, cisplatin can also serve as an artificial enzyme to generate H_2_O_2_ as the reactant of CDT through cascade reactions, which remarkably enhances the anticancer effect of nanomedicine. The imaging function of nanomedicine can also be successfully introduced by doping PTCG NPs with Gd to trace the delivery route and drug release behaviors of nanomedicine, achieving precise diagnosis and treatment of cancer (Ren et al., 2020).

## *Cancer* Theranostic Probes for Gene Therapy

Gene therapy has enormous potential for the treatment of various diseases due to its highly specific and effective regulation of gene and protein expression, which introduce exogenous nucleic acids such as genes, gene segments, oligonucleotides, miRNAs, or siRNAs into targeted tumor cells, correcting the abnormally expressed genes and restoring their normal biological function, so as to achieve the purpose of treating diseases (Shu et al., 2014; Wang et al., 2016; Zhou et al., 2017; Jiang et al., 2019; Boumahdi and De Sauvage, 2020; Wang and Yu, 2020). This treatment approach is apparently more suitable for cancer since the successful transfected cancer cells have a proliferative advantage and can further amplify the gene therapy effect through division process. In the case of systemic and presystemic deliveries, the administration of naked nucleic acids is largely hindered by biological barriers, nuclease decomposition, renal clearance, or immune response (Han et al., 2013; Kim et al., 2016). To overcome these limitations, nano-carriers/vectors that can protect the nucleic acid cargo from damage and ensure the efficient targeting of these therapeutic nucleic acid into the tumor cells to carry out the desired clinical results and decrease side effects are used (Yang et al., 2014). In combination with the imaging ability of the nanoformulations, the theranostic probe can not only accurately detect the tumor, but also reflect the process of *in vivo* behavior of target gene such as pairing, silencing, or transfection in the treatment process, enabling visual assessment of efficacy (Wolfbeis, 2015).

### Nanotechnology Improves Viral Mediated Gene Delivery for Tumor Theranostics

Viral systems, the earliest gene carriers, are widely accepted as efficient gene delivery tools and have an excellent transfection efficiency (Kotterman et al., 2015; Ni et al., 2016). Viruses have the innate ability to break through the biological barriers in the body and specifically target the host cells and begin replicating, which enables them to protect their genes from nuclease attack to safely enter the nucleus and effectively integrate into the host genome and realize a high gene expression (Lundstrom, 2003; Liu and Deisseroth, 2006; Waehler et al., 2007; Wu and Zhou, 2011). Using these properties, viruses are utilized to selectively target tumor cells and deliver the gene with higher efficiency (Koppers-Lalic et al., 2013). However, viruses have crucial setbacks that largely limit the *in vivo* applications. The randomness and uncontrollability of viruses to re-combinate the tumor may lead to severe side effects, which has aroused certain solemn concerns (Santiago-Ortiz and Schaffer, 2016; Tian Y. et al., 2018; High and Roncarolo, 2019). Thus, nanotechnology has attempted to address these problems in different ways.

Liu and coworkers developed an oncolytic adenovirus (OA) delivery platform that can be employed for accurate virotherapy of tumors. Such a platform is constructed by coating OA with bioengineered cell membrane nanovesicles (BCMNs), which is useful for the specific targeting of tumor cells and decreases the antiviral immune response of the body. The tumor targeting ligands can be inserted into the BCMNs, which not only endow the nano-platform with the ability of tumor targeting, but also provides a stealth effect under immune surveillance. In addition, this targeted delivery process can be monitored by *in vivo* fluorescence imaging. Through these features, OA can offer systemic delivery efficiently with immune escape ability and few side effects (Lv et al., 2019). In another study, Xie and coworkers proposed an approach to protect viruses from removal by the host immune system and prolong their *in vivo* retention to enhance the tumor targeting. They developed an engineered OA by enwrapping them with a calcium and manganese carbonates (MnCaCs) biomineral shell. After arriving at the tumor sites, MnCaCs will dissolve under the acidic condition and release Mn^2+^, which can not only catalyze the endogenous H_2_O_2_ into O_2_ to accelerate the replication of OA, but also enhance the signal of both MRI and PAI, providing real-time monitoring for the therapy. This theranostic approach demonstrated the promise for visualized oncolytic virotherapy with high efficacy by systemic administration (Huang et al., 2019).

Nanotechnology can offer new avenues in virus-based gene therapy; nevertheless, the size limitation of the transgene that can be packaged into a viral vector and the technical problems faced by the large-scale production of viral vectors still make it difficult for viral-mediated gene delivery to achieve clinical translation.

### Nano-Based Nonviral Gene Delivery for Tumor Theranostics

Owing to the safety concerns of viral-mediated gene delivery, currently, several other carriers have been implemented as an alternative to viral vectors, especially for engineered nano-vectors that could mimic the features of viral vectors as a type of novel gene carrier (Fenton et al., 2018; Yuan et al., 2019).

Liposome-based materials have been studied extensively for the delivery of nucleic acids, as they can carry various types of nucleic acids, such as mRNA and siRNA, within a hydrophilic cavity (Ozpolat et al., 2014; Mangraviti et al., 2015; Duro-Castano et al., 2017; Kuang et al., 2017). For example, Anderson and coworkers developed a combinatorial library of ionizable lipid-like materials by using a three-dimensional multi-component reaction system to construct mRNA delivery vehicles that carry mRNA to the desired area *in vivo* and trigger an immune reaction. They found that several formulations of this library can successfully induce a strong immune response, and can suppress the tumor growth, thereby prolonging the survival rate of tumor-bearing mice through mRNA delivery (Miao et al., 2019). The liposomes can be modified with targeting molecules to endow them with the ability to recognize the lesions specifically. For example, Zhao and coworkers developed a PEGylated liposome loaded with pcDNA3.1-CSF1-mES for gene therapy of vascular endothelial cell and tumor imaging, which conjugated with anti-CD105 mAb to obtain targeting ability. With this targeting moiety, the liposomes can successfully recognize the tumor-derived endothelial cells *in vivo*, monitored by fluorescence imaging of tumor-bearing mice, which can significantly improve the gene expression and thereby inhibit the tumor growth (Zhuo et al., 2018). Such results demonstrate the advantages of the liposomes to enhance tumor targeting, imaging, and gene transfer applications.

One another advantages of using liposomes to deliver nucleic acid is that they facilitate cellular uptake through interaction with lipids in the cell membrane to induce membrane fusion, which can directly release the nucleic acid in the cytoplasm to avoid trapping in endosomes, or by inserting the targeting molecules that enhance the specific binding ability to cell receptors (Alex and Sharma, 2013). Kim and coworkers employed porous silicon nanoparticles as the siRNA host, which were then encapsulated by the fusogenic lipid with the targeting peptide modified on the surface for selective tissue homing. Importantly, the fusogenic lipid coatings can induce the membrane fusion between the liposomes and the cellular membrane, which efficiently deliver the siRNA into the cytoplasm to avoid endocytosis. In an ovarian-cancer- tumor-bearing mouse, this liposome can successfully target the tumor area shown by *ex vivo* fluorescence imaging, efficiently silence the Rev3l subunit of polymerase Pol ζ to inhibit DNA repair in combination with cisplatin, and reprogram tumor-associated macrophages (TAMs) into a proinflammatory state (Kim et al., 2019).

However, liposome carriers still face some problems. The interactions between lipid and cell membrane help the nucleic acid enter the cells, but also has potential toxicity (Xue et al., 2014), and some liposomes have been found to cause an immune response that leads to immunotoxicity (Vangasseri et al., 2009; Ni et al., 2016). Thus, the biochemical studies of these liposomes should be considered more carefully when they are used as carriers.

**FIGURE 3:**
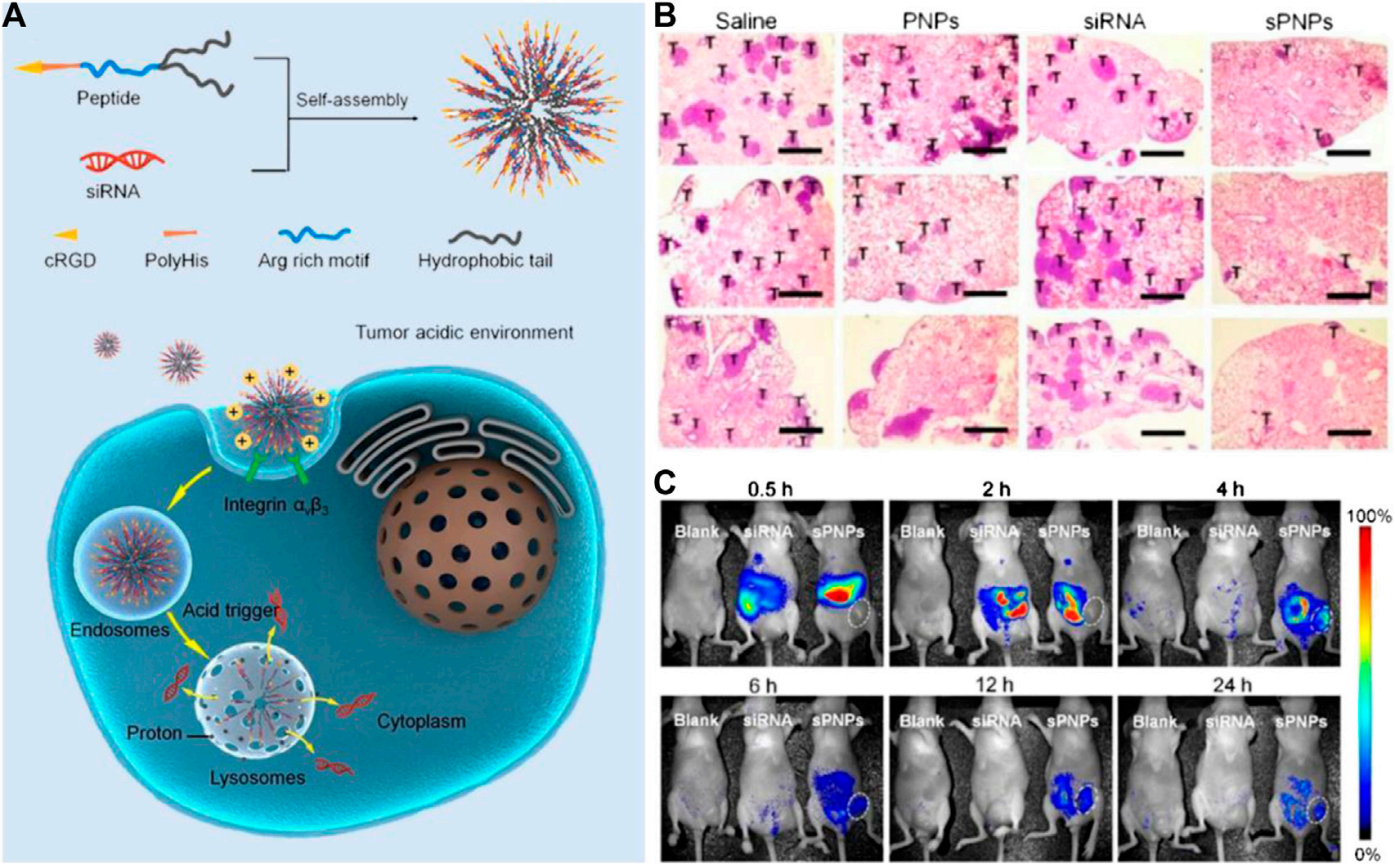
**(A)** Schematic of the self-assembly of the sPNPs nanoparticles and their antitumor mechanism. **(B)** Effects of PNP, free TF siRNA, and sPNPs on metastasis in 4T1 tumor bearing mice. **(C)** *In vivo* fluorescence imaging of MDA-MB-231 tumors bearing mice after administration of nothing **(left)**, free Cy5-siRNA **(middle)**, or Cy5-sPNPs **(right)**. Reproduced with permission from Liu S. et al. (2019). Copyright 2019 American Chemical Society.

**FIGURE 4:**
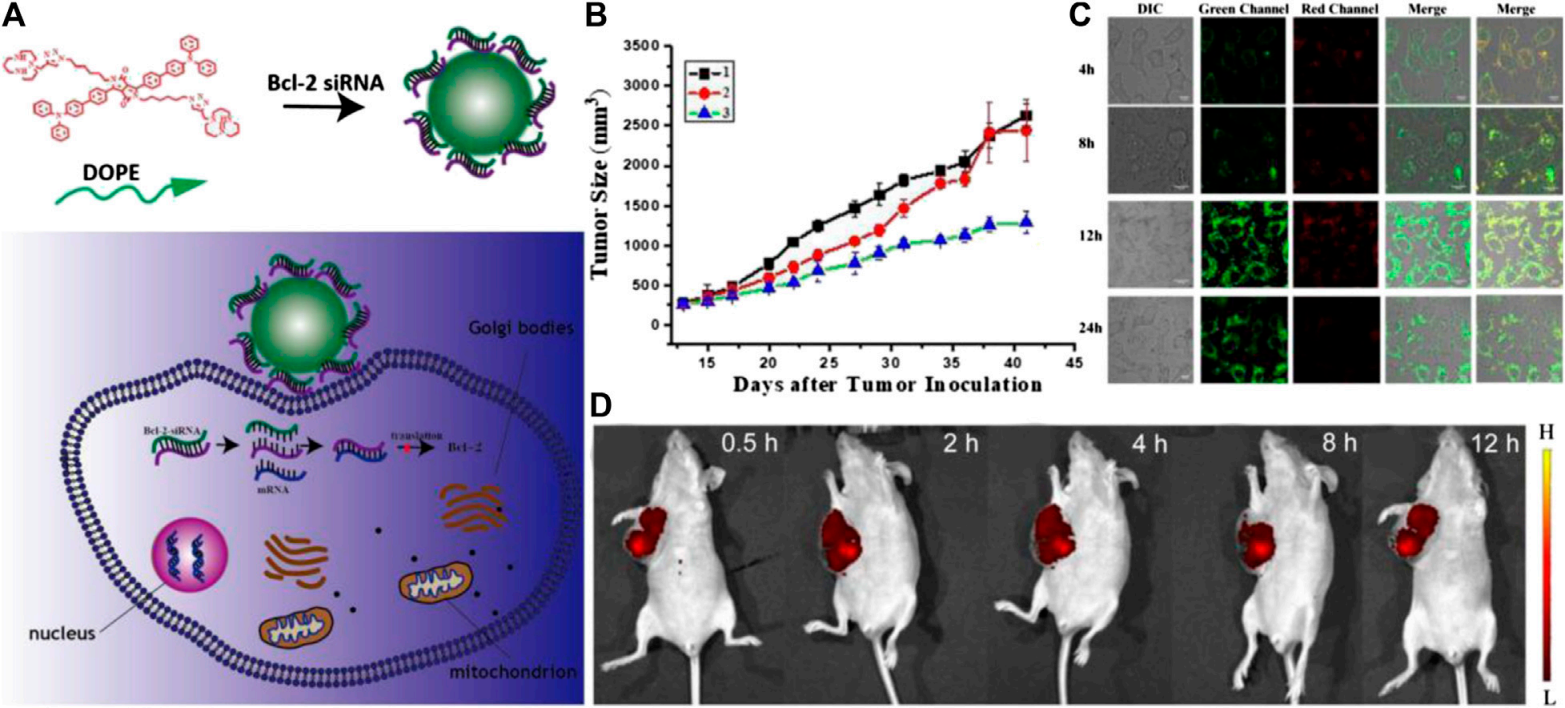
**(A)** The scheme of tumor targeting gene vectors for visual tracking of Bcl-2 siRNA transfection and anti-tumor therapy. **(B)** Body weight changes of the nude mice of different groups over 41 days; **(C)** Fluorescent confocal laser scanning microscopic images of Hela cells after treatment with DPL/siRNA under different incubation time. The siRNA was labeled with fluorescein amidite (FITC). The embedded scale bars correspond to 10 μm. **(D)** Fluorescence imaging of tumor-bearing mice *in vivo* after DPL/siRNA injection via tail vein. Reproduced with permission from Sun et al. (2020). Copyright 2020 American Chemical Society.

In addition to liposomes, other types of nanomaterials have been employed to serve as carriers of nucleic acids, which can realize the theranostic treatment of tumors (Guo and Huang, 2014; Tsouris et al., 2014). For example, Tang and coworkers designed a series of Ag@AIE core@shell nanocarriers with tunable and uniform morphology by employing of aggregation-induced emission luminogen (AIEgen). This nanocarrier successfully facilitated the delivery efficiency of siRNA, which endowed this nanocarrier with excellent capabilities in specific mRNA interference and tumor growth suppression. In addition, the cellular uptake, endosomal escape of siRNA, and tumor area can be visualized real-time by the unique AIE fluorescence signal of nanocarriers *in vivo*. Compared to the commercial transfection reagents, the biocompatibility, delivery efficiency, and reproducibility of such nanocarriers are significantly improved, which represent a promising future in RNAi-related cancer therapy (He et al., 2019). In another study, Nie’s group reported a novel peptide-based nanoparticle that can specifically deliver and release the small interfering RNA (siRNA) into the solid tumor to silence the expression of tumor-associated TF, a major initiator of blood coagulation that played a critical role in the hematogenous metastasis of tumors (Figure 3). With the ability of tumor targeting and prohibition of tumoral TF expression, such nano-systems can effectively inhibit the interactions of circulating tumor cells with platelets and reverse the tumor coagulant state. Furthermore, the nano-system can also prevent the spreading of tumors, which significantly inhibits metastasis to the lung in a mouse breast cancer model through attenuating TF expression of tumor cells. In addition, after labeling the siRNA of nanosystems with Cy5, the *in vivo* fluorescence imaging indicated that it can efficiently accumulate in the tumor region, which ensures the local concentration of siRNA to improve the efficacy. This nano-system displays a novel approach to safely and precisely knock down tumor-associated TF for preventing metastasis and may offer a new form of tumor gene therapy (Liu S. et al., 2019). Sun and coworkers designed and synthesized a tumor-targeting fluorescent gene vector (DPL), which was derived from diketopyrrolopyrrole (DPP) via the modification with aneN_3_ and TPA units (Figure 4). Owing to the siRNA condensation capabilities, DPL can successfully in real-time display the process of cellular uptake and siRNA release *in vitro* and can kill the cancer cells by carrying a small amount of siRNA. On an animal level, DPL can recognize tumor regions in mice through the EPR effect after intravenous injection and deliver the Bcl-2 siRNA for tumor treatment, which can be monitored by fluorescence imaging *in vivo*. This work provides a new non-viral gene vector for achieving real-time tracking of the therapeutic siRNA delivery process as well as cancer therapy (Sun et al., 2020).

Polymers can also be employed as a class of potential carriers to load nucleic acid. Wang and coworkers designed a multifunctional diblock copolymer and constructed it into heterogenous membrane polymersomes with “boarding” and “debarkation” gates, which can act as gene vectors for encapsulating nucleic acid in an aqueous solution through opening the “boarding” gate, and then releases them by triggering the “debarkation gate” using the proton sponge effect within the target cells. The encapsulated plasmid DNA exhibit better transfection ability than the adsorbed plasmid DNA due to the more excellent protection for plasmid DNA available inside the polymersomes. Through the fluorescent imaging *in vivo*, the signal of GFP-encoding plasmid DNA can be seen 6 days post-injection, confirming that the polymersomes increase the transfection and expression of pDNA in animals (Wang et al., 2018). Lee and coworkers developed a biocompatible poly (D, l-lactide-glycolide) (PLG) nanoparticle with imaging function and therapeutic genes, which was modified with rabies virus glycoprotein (RVG) peptide (RVG-PNPs) to target neuroblastoma. As a result, RVG-PNPs had great potential and can be used as cancer detection tools for molecular imaging in a neuroblastoma-bearing mouse model. Compared with unmodified nanoparticles (PNPs), the RVG-PNPs can significantly enhance cell uptake and gene silencing of N2a cells *in vitro* and can effectively fight against neuroblastomas specifically through a therapeutic gene cocktail (siMyc, siBcl-2, and siVEGF) *in vivo*. With fluorescence imaging, these nanoparticles precisely detect the neuroblastoma of mice (Lee et al., 2016).

These above works show the great potential of a plenty of nano-agents to serve as carriers to deliver nucleic acids, including various DNA, siRNA, or mRNA, with tumor visualization function, which provides a new avenue for tumor diagnosis and treatment.

## Theranostic Probes for Cancer Immunotherapy

Immunotherapy is a kind of treatment approach based on the natural function of the immune system in protecting the host. Its cardinal features including potency, specificity, and memory. In recent years, cancer immunotherapy has made great success in clinic, with chimeric antigen receptor (CAR) T-cell therapies and checkpoint blockade treatment with immunomodulatory agents that block the inhibitory receptors cytotoxic T lymphocyte antigen-4 (CTLA-4) or programmed death-1 (PD-1) (Fridman et al., 2012; Bakos et al., 2018) the most common options. However, as the immune drugs activate the immune system, they may also lead to severe nonspecific systemic inflammation and autoimmune side effects, resulting in a series of adverse reactions from the body, including significant weight loss, systemic cytokine storms, and even death (Grahnert et al., 2011; Weber et al., 2015).

Currently, several formulations based on nanotechnology are being explored to prepare anti-tumor immune preparation, which possess some degree of intrinsic adjuvant or immunostimulant properties compared with conventional low-molecular-weight immune agents and should also be able to co-encapsulate multiple antigenic epitopes, targeting ligands and external adjuvants into a single carrier. These nano-based anti-tumor immune agents are expected to not only protect the structural integrity of the antigens, effectively deliver them to the desired sites, and make these substances synergistic to induce an immune response, but also reduce the systematic side effects owing to their specific targeting ability (Liu et al., 2014; Song W. et al., 2019).

Based on the different targets of nano-based immune agents and distinct pathways of immune response they induced to kill tumor cells, in this section, two major targeting directions are being explored, in which nanomedicines are designed to directly target the tumor immune microenvironment or to target the peripheral immune system. Meanwhile, the imaging function of nano-agents endows them with the powerful ability to track and monitor the immunotherapy process, which not only ensures the accuracy of treatment, but also provides more guidance information for prognosis evaluation to avoid immune side effects to the greatest extent (Torrente-Rodríguez et al., 2020).

### Target Peripheral Immune Cells for Tumor Theranostics

Peripheral immune cells are a class of immune cells including lymphocytes, antigen presenting cells (APCs), mast cells, and other immune cells, which reside in the peripheral immune organs, and are mainly responsible for recognizing antigens and participating in the immune response. Within the peripheral immune organs, the induction of anti-tumor immunity is a multi-step process, which includes the recognition and presentation of tumor antigen by APCs, activation of antigen-specific T cells, and attacking of the tumor cells by effector T cells (Song Q. et al., 2019). However, tumor cells can usually escape immunosurveillance through a number of special mechanisms, which largely reduce the body's immune response and ensures the survival of cancer cells (Mohme et al., 2017). In order to enhance the specific tumor cell immune response, nanoformulations have been designed as regulators to promote the maturation of dendritic cells and the activation of T cells.

Compared with the conventional low-molecular -weight immunoregulators, the well-engineered nanomaterials can not only enhance the power of immune response via intrinsic immunogenicity, but can also track the migration routes of immune cells after activation. Liu’s group loaded ovalbumin (OVA) antigen on the upconversion nanoparticles (UCNPs), which can be efficiently engulfed by dendritic cells (DCs), to label them with upconversion luminescence (UCL) and induce the maturity of DCs *in vitro* as an artificial DCs vaccine. After intradermal administration at the right footpad of such an artificial DCs vaccine, their homing behavior to draining lymph nodes can be monitored *in vivo* through UCL imaging. Importantly, the strong antigen-specific immune responses of such a nanovaccine, including enhanced T cell proliferation, interferon gamma (IFN-γ) production, and cytotoxic T lymphocyte (CTL)-mediated responses, are induced by this nanoparticle-pulsed DCs vaccine, which effectively suppresses the tumor growth (Xiang et al., 2015).

Apart from the artificially modified APCs *ex vivo*, several nano-agents have been designed to directly activate the APCs *in vivo* to reduce production costs, simplify the preparation procedure, and make large-scale production easier. To enhance the activation efficiency of APCs *in vivo*, antigens and adjuvants (mainly a class of receptors agonists) are usually co-delivered by nano-systems. For example, Liu and coworkers reported a kind of cancer nanovaccine synthesized using nanoscale coordination polymer (NCP) composed of Mn^2+^ ions and a nucleotide oligomerization binding domain one agonist, meso-2,6-diaminopimelic acid (DAP), as the organic ligand, to load OVA antigen. The obtained OVA@Mn-DAP nanovaccine could actively migrate into lymph nodes after local administration, as monitored by MR and optical imaging *in vivo*. Therefore, such a nanovaccine can be used as an effective tumor vaccine to accelerate the maturation of dendritic cells (DCs) via stimulating the Nod1 pathway with DAP, thereby promoting the cross-presentation of antigens (Zhao H. et al., 2019).

**FIGURE 5:**
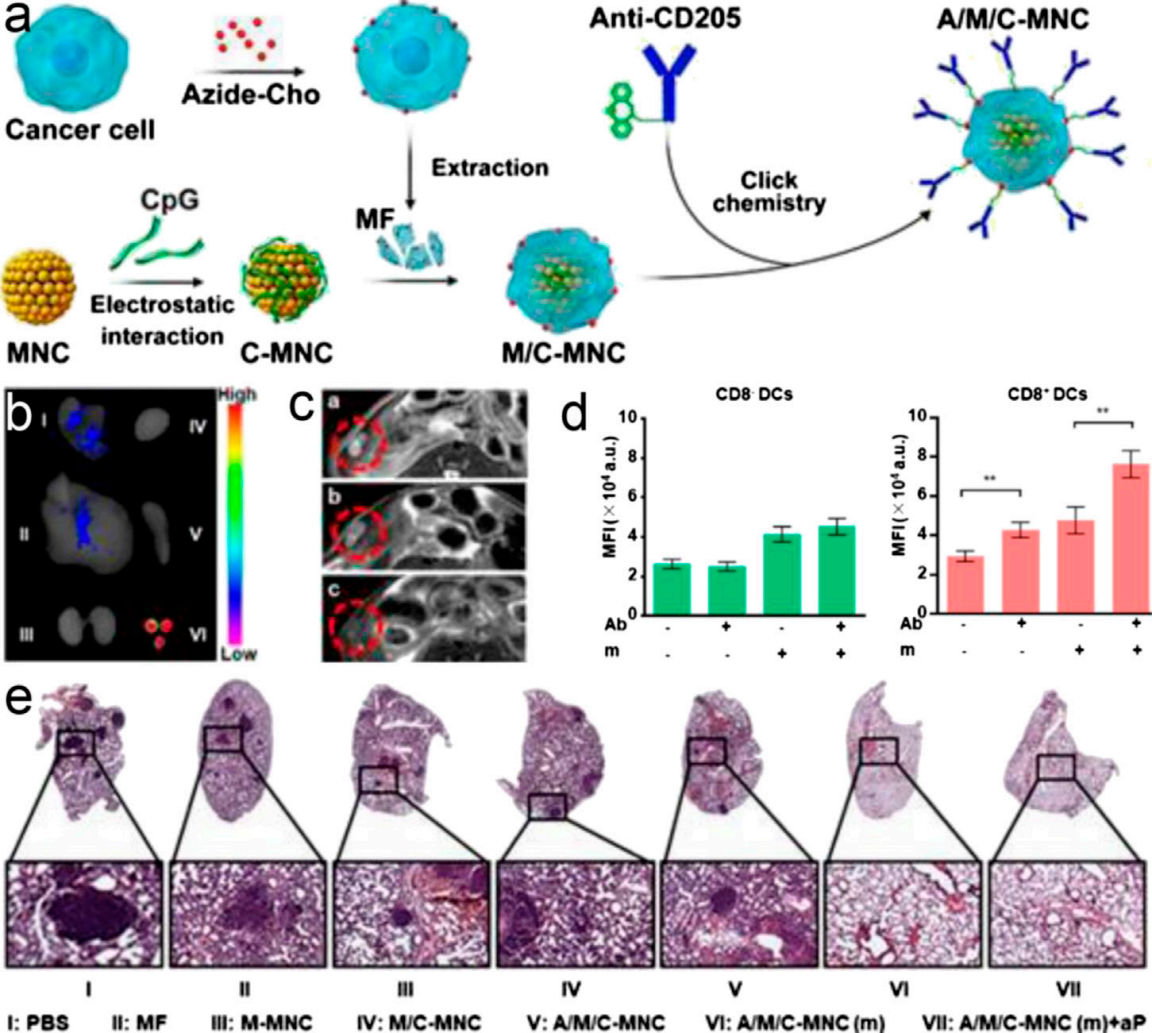
**(A)** Fabrication process of A/M/C-MNC. **(B)** Fluorescence imaging of excised lymph nodes and visceral organs two days after vaccination with A/M/C-MNC. I-VI were lung, liver, kidneys, heart, spleen, and lymph nodes, respectively. **(C)** *In vivo* *T*_2_-weighted MR imaging of mice vaccinated with different formulations. The red circles outline the lymph nodes. **(D)** MFI of DCs in the lymph nodes after vaccination with different DiD-labeled formulations. Ab, anti-CD205 conjugation; m, magnetic field retention in the lymph nodes. **(F)** H&E-stained lung sections of different groups. All data represent the mean ± SD (n = 6). Reproduced with permission from Liu S. et al. (2019). Copyright 2019 American Chemical Society.

Due to the strong escape mechanism of cancer cells, the tumor suppressive ability of effector T cells activated by a single antigen is still insufficient. Tumor vaccines are expected to contain a variety of effective tumor antigens to activate multiple antigen-specific T cells through more APCs to achieve better tumor treatment effects (Chang et al., 2020). In this regard, tumor cell membranes, which express multiple tumor-associated antigens, are becoming a potential candidate to construct nanovaccines with multi-antigens. Li and coworkers have developed a novel class of magnetosomes through coating TLR agonist CpG-ODN and Fe_3_O_4_ nanoparticles with anti-CD205 mAb modified cancer cell membranes. The Fe_3_O_4_ nanoparticles endow the magnetosomes with magnetic retention ability in the lymph nodes, as revealed by MRI, which prolong the time period for antigen uptake by DCs. Moreover, the cancer cell membrane provides various cancer specific antigens for subsequent multi-antigenic responses. In addition, the anti-CD205 mAb can promote the uptake of CD8^+^ DCs, thus facilitating MHC I cross-presentation. Through tail base subcutaneously injection of the nanovaccine and attraction of the magnetic field at inguinal lymph nodes, the *in vivo* T cells can be activated with satisfied clonal diversity and superior cytotoxic activity, leading to great prophylactic and therapeutic effects of cancer (Li et al., 2019) (Figure 5). Du and Li developed an intelligent clustering nanoparticle delivery system, iCluster, whose size can change from ∼100 to ∼5 nm in the acidic tumor microenvironment, thereby improving the implantation of particles in the interstitium of the primary tumor. Furthermore, the resultant smaller nanoparticles could drain into lymph nodes through tumor lymphatics, thus inhibiting tumor metastasis (Liu J. et al., 2019). In another work, Grippin designed a kind of multifunctional magnetic liposome loaded with RNA to trigger the antitumor immune response as an early biomarker of cancer treatment. These particles can induce the maturity of DCs more effectively than electroporation, thereby successfully suppressing the tumor growth in mice treatment models. In addition, the iron oxide nanoparticles have been proven to promote the transfection of DCs, and trace the migration path of DCs through MRI (Grippin et al., 2019). Tan’s group developed a novel intratumoral nano-agents delivery system for long-term suppression of tumors through apoptotic bodies (AB) of cancer cells as the drug carriers. Specifically, CpG immunoadjuvant-modified gold-silver nanorods (AuNR-CpG) was encapsulated by AB to construct the nanomedicine. After intravenous injection, these nano-agents can be selectively ingested by inflammatory Ly-6C^+^ monocytes, which then actively penetrate into the deep solid tumor via their natural tumor-homing ability, which can be indicated by the fluorescent imaging. In addition, AuNR-CpG/AB could effectively induce the maturity of DCs *in vivo* under NIR irradiation, thereby promoting the secretion of the proinflammatory cytokine by DCs to activate effector T cells, thus eliciting a potent immune response to fight against the tumors. Such cell-mediated delivery systems can not only successfully inhibit the primary tumors, but also elicit a great immunity to prevent tumor metastasis and recurrence (Zheng et al., 2020).

The above pre-clinical studies demonstrated the great potential of nano-agents in cancer immunotherapy through initiating an effective and durable antitumor response. However, the limitations of antigen identification, APC activation efficiency, and weak immune response still limit the practice of cancer nanovaccines (Bol et al., 2016; Milling et al., 2017; Sprooten et al., 2019). Larger animal models and larger amounts of validation results are required to promote the clinical translation, which will also help to further determine the precise modalities of the application.

### Target the Tumor Immune Microenvironment for Tumor Theranostics

The clinical successes in immunotherapy have been astounding, but also unsatisfactory. Immune-checkpoint blockade approaches, such as CTLA-4 and PD-1, which are commonly used in clinical trials, are unfortunately ineffective for a large number of patients, who do not respond to the triggers. Recently, several pieces of research have shown that the tumor microenvironment is one of the major barriers to effective treatment of carcinoma through immunotherapy (Fu et al., 2020). Within the tumor microenvironment, a class of immune or non-immune cells are found, which can secrete various factors, leading to a chronic inflammatory and immunosuppressive environment, in which cancer cells can adapt and proliferate comfortably without eradication by host immunosurveillance (Gajewski et al., 2006; Binnewies et al., 2018). To overcome this obstacle, a nano-based immunomodulatory agent that can directly target the immune microenvironment in solid tumors has been developed, which can monitor and mediate the immunosupressive state, or even remodel the microenvironment to improve the tumor immunotherapy.

#### Enhance the Killing Effect of Immune Cells for Tumor Theranostics

NK cells and T lymphocytes have the ability to fight against tumors, however, they are usually suppressed by the tumor immune microenvironment (Whiteside, 2008). Based on this phenomenon, several nano-agents have been designed to help these immune cells break through the barriers and recover the ability to kill tumors.

Chen and coworkers reported an inherently therapeutic fucoidan-dextran-based magnetic Nanomedicine (IO@FuDex3) connected with antibodies, including checkpoint inhibitors (anti-PD-L1) and T-cell activators (anti-CD3 and anti-CD28). Such IO@FuDex3 can remodel the immunosuppressive tumor microenvironment by directly inducing T-cell activation and block the immunosuppressive PD-L1 pathways via intravenous medication. Moreover, the magnetic navigation effectively amplified the accumulation of IO@FuDex3 at the tumor site and minimized the systemic exposure, which can be clearly visualized by single-photon emission computed tomography imaging, minimizing the off-target side effects (Chiang et al., 2018). In another work, Nie prepared magnetic nanoclusters (NCs) modified with responsive PD-1 antibody by using a pH-sensitive benzoic−imine bond and inverse electron-demand Diels-Alder cycloaddition. Owing to the high magnetization and superparamagnetism of NCs, the T cells, together with PD-1 antibody, can be magnetically attracted within the tumor area under the monitoring of MRI. Arriving at the tumor sites with a lower pH, the benzoic−imine can be hydrolyzed, causing the release of PD-1 antibody, which can therefore be coupled with the adoptive T cells to activate their therapeutic effects. This study successfully combined the immune cytotoxicity and checkpoint blockade together in a synergistic manner (Nie et al., 2019).

However, owing to the diversity of immunosuppressive factors in the tumor microenvironment (TME), the immunosuppression of solid tumors is too strong to overcome and the amount of antigen-specific T cells in the microenvironment is not enough. These limitations largely weaken the effect of tumor treatment, and hinder the clinical application of this strategy of immunotherapy (Song C. et al., 2019). It may be possible to consider the possibility of combining this approach with other therapies in the future.

#### Attenuate the Inhibition of Immune Cells for Tumor Theranostics

Tumor-associated macrophages (TAMs) are a class of major immunosuppressive players in the tumor immune microenvironment, which can mainly be polarized into anti-tumor M1 type and pro-tumor M2 type under the action of different stimulating factors (Ovais et al., 2019). Although M1 phenotypes can inhibit the tumor growth and can be seen as a good hallmark of prognosis, they are usually suppressed in the microenvironment. On the other hand, M2 phenotypes of TAMs support the tumor growth via secreting a variety of cytokines, such as IL-10, and are usually active in the tumor microenvironment due to tumor stimulations (Hattermann et al., 2014). They also promote angiogenesis by secreting corresponding hormones, promote the formation of an immunosuppressive microenvironment, and accelerate the growth of tumors. In established human progressive tumors, TAM usually expresses an M2 phenotype, thereby promoting tumor progression, metastasis, and resistance to chemotherapy (Sousa et al., 2015). For this reason, a nano-based immunomodulator that can inhibit or modify the TAMs has been designed to increase the efficacy of cancer immune therapy. For example, Fan and coworkers developed an elaborate ferrimagnetic vortex-domain iron oxide nanoring and graphene oxide (FVIOs-GO) hybrid nanoparticle, which exhibited high thermal conversion efficiency under alternating magnetic field (AMF) and could generate a large amount of ROS under alternating magnetic field, inducing a potent immune response in a hypoxic tumor microenvironment. In addition, the in-situ heat can be generated at a physiological tolerable temperature through magnetothermodynamic therapy, which can be guided by *T*_2_-weight MR imaging *in vivo*. Importantly, such nanoparticles can promote the macrophages’ polarization to pro-inflammatory M1 phenotypes, and further elevate the tumor-infiltrating T lymphocytes to kill tumor cells (Liu X. et al., 2020). In another work, Weissleder and coworkers prepared R848-loaded β-cyclodextrin nanoparticles (CDNP-R848), which can successfully deliver the drug to TAMs effectively. Such nanoparticles can remodel the tumor immunosuppressive microenvironment toward an M1 phenotype, in order to prohibit the tumor growth and prevent the metastasis and recurrence of the tumor. With the *in vivo* imaging of an orthotopic lung adenocarcinoma mice model, R848 and the CDNP carrier can be co-localized *in vivo* at the subcellular level within TAMs, which confirmed that the CDNPs successfully deliver R848 to TAMs. By combining with the immune checkpoint inhibitor anti-PD-1, the strength of the immunotherapy response can be further increased. This study displayed the satisfactory ability and efficacy of nano-agents designed for TAMs remodeling for cancer immunotherapy (Rodell et al., 2018).

In addition to TAM, myeloid-derived suppressor cells (MDSCs) are another kind of immunosuppressive cells involved in the formation of the tumor immune microenvironment, which have also been seen as an available targeting marker for alleviation of the immunosuppression state of a tumor microenvironment. MDSCs often suppress the immune function of T cells and NK cells in a tumor microenvironment, and establish the pre-metastasis microenvironment via promoting angiogenesis and recruiting and interacting with other immunosuppressive cells such as M2 phenotype TAMs and Tregs (Uhel et al., 2017; Han et al., 2018). In order to inhibit the activity of MDSCs, Yu and coworkers developed a BSA-based nano-regulator incorporating MnO_2_ and PI3K γ inhibitor IPI549 to reshape the tumor immune microenvironment to unleash the immune system. The intravenously injected nano-regulators effectively accumulated within the tumor tissue to relieve hypoxia via oxygen generated by MnO_2_, and down-regulated the expression of immunosuppressive PD-L1. The binding of released IPI549 to PI3K γ on MDSCs can successfully cause the M1-polarization of TAMs and re-activate the tumor-suppressive T-lymphocytes to kill tumor cells. This process can be visualized by the tumor-specific MRI induced by local generation of Mn^2+^ in the tumor microenvironment (Yu et al., 2019).

### Directly Target Cancer Cells for Tumor Theranostics

**FIGURE 6:**
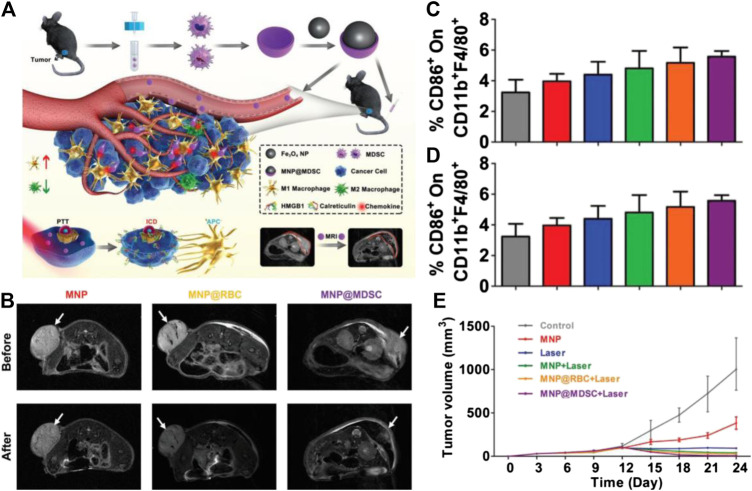
**(A)** Schematic illustration of the synthesis of MNP@MDSC and its application in cancer theranostics **(B)** *T*_2_-weighted MR images of B16/F10-tumor-bearing mice taken before and after injection of the nanoparticles. **(C)** Percentage of CD86 ^+^ cells within the CD11 b + F4/80 + population of suspension cells from the mouse spleen after different treatments. **(D)** Percentage of CD206 + cells within the CD11b + F4/80 + population of suspension cells from the mouse spleen after different treatments. **(E)** Growth (in volume) curves of mouse tumors in different treatment groups. Reproduced with permission from Zhang F. et al. (2019). Copyright 2018 Wiley-VCH Verlag GmbH & Co. KgaA.

As the tumor cells are suffering from apoptosis, they will over-express several membrane proteins, which can change their state from non-immunogenic to immunogenic, and consequently stimulate the anti-tumor immune effect in the body, known as the immunogenic death (ICD) of tumors (Yatim et al., 2017; Irvine and Dane, 2020). Interestingly, this strategy of tumor treatment leads to a “chain reaction” of tumor death, that is, the first apoptotic tumor cell initiates the chain initiation step of cell death, which can provide the antigens to APCs, and then these antigens will be presented to the cytotoxic T lymphocytes to further induce more tumor cells apoptosis, as in the propagation step. Nano-agents can be designed to be the initiator of this chain reaction and/or the promotor to magnify the immune response. For example, Na developed the biocompatibility controllable immuno-sensitizer (BCI) based on polyethylene imine with optical imaging function and pH response ability, which was successfully used in tumor theranostics. After arriving at tumor sites, BCI can induce the necrosis of tumor cells, which subsequently leads to the release of a variety of tumor antigens. Such antigens will then be recognized by antigen presenting cells such as DCs in tumor draining lymph nodes and be presented to recruite cytotoxic T cells to attack the tumors (Shin and Na, 2019). Similarly, Xie and coworkers constructed magnetosomes by coating both Fe_3_O_4_ nanoclusters (NCs) and TGF-β inhibitors with artificially engineered leukocyte membranes, in which PD-1 antibodies are employed to anchor on the surface of leukocyte membranes. Through intravenous medication, the camouflage of the leukocyte membrane can prolong the blood half-live of this nanostructure, and the NCs core within the membrane endow the magnetosomes with enhanced MRI ability. Upon arriving at the tumor sites, PD-1 antibodies and TGF-β inhibitors synergistically create an immunogenic microenvironment, thereby increasing the percentage of M1 macrophages to produce H_2_O_2_ and simultaneously initiate the Fenton reaction through the catalysis of Fe^3+^ released from NCs. The accumulated ROS will subsequently activate the ferroptosis process of cancer cells, which can then produce a large amount of tumor antigens and increase the immunogenicity of the tumor microenvironment to amplify the immune response. As a result, such synergistic immunomodulation and ferroptosis leads to more satisfactory tumor therapeutic effects (Zhang F. et al., 2019). In addition, Yu and coworkers extracted the cell membrane of MDSCs to coat the iron oxide nanoparticles, which displayed a superior ability to escape immune surveillance, precisely detect the tumor through MRI, and treat the tumor by PTT. In comparison with the red blood cell membrane-coated nanoparticles (MNPs@RBC) or naked MNPs, MNP@MDSCs possess high selectivity in tumor targeting. Upon arriving at the tumor regions, MNP@MDSC can first act as a PTT agent to initiate the antitumor immune response through inducing ICD of the tumor, then they reprogram the TAM to reduce the tumor metabolic activity, further promoting the immunotherapy of the tumor (Yu et al., 2018) (Figure 6).

In addition to inducing tumor cell death to express immunogenic protein molecules, theranostic nanoprobes are also enabled to directly enhance the expression of the immunogenic protein of tumor cells. Dravid and coworkers reported a high-density lipoprotein (HDL) mimicking magnetic nanostructures (HDL-MNSs), which can selectively bind with the HDL receptor, scavenger receptor type B1 (SR-B1), thereby causing cellular cholesterol depletion via hindering the cholesterol secretion in SR-B1 receptor positive lymphoma cells. In addition, the thermal activation of MNS core under an external radio frequency field can lead to antitumor immune responses by increasing the heat shock proteins’ expression to promote the maturity of APCs and realize lymphocyte trafficking. Besides, HDL-MNS with *T*_2_ enhanced ability and specificity toward the SRB1 receptor cause a distinguishable contrast between SR-B1 positive and negative cells, which highlight its capability in molecular imaging (Singh et al., 2019).

Although the above studies show the promising effect on tumor theranostics, the immunosuppressive microenvironment of solid tumors is still a big obstacle limiting tumor immunotherapy. Considering that tumors can assimilate the surrounding immune cells, which subsequently facilitates the proliferation and invasion of cancer cells, more strategies should be proposed to suppress and even reverse these behaviors.

## Theranostic Probes for Physical Therapy of Tumors

In addition to chemotherapy, immunotherapy, and gene therapy, physical therapy is another class of treatment method to deal with cancer in clinical application. Compared with other treatment approaches, physical therapy that treats the disease through physical means such as thermotherapy, photodynamic therapy, or radionuclide therapy, can not only kill the tumor cell with high efficiency, but also minimize the side effects through reducing systematic exposure, thereby improving the quality of life of patients (Dougherty et al., 1998; Agostinis et al., 2011). The rich intrinsic physical and chemical properties of nanomaterials have made them potential candidates for the tumor theranostic probes involved in physical therapy. Compared with traditional physical therapy, nano-based physical therapy can not only significantly improve the efficiency of tumor therapy via passive targeting ability through EPR effect, but can also provide highly specific, robust detection of tumors in high contrast images to guide the physical stimuli.

### Photothermal Nanoprobes for Tumor Theranostics

Photothermal therapy (PTT) is a kind of non-invasive treatment which relies on an external laser source to make the photothermal agents absorb photon energy and convert it into heat energy, and thereby cause thermal ablation of tumor cells (Pan et al., 2019). This technique of thermotherapy of tumors has been widely adopted in clinical treatments (Liu Y. et al., 2019a). But the efficiency is not so ideal in treating the tumor tissues growing deep in the body due to the limitation of light penetration depth. In comparison with conventional clinical photothermal agents, the wavelength of the excitation source of nanomaterials with NIR absorption can be adjusted in the NIR region, which largely increases tissue penetration. At the same time, through combining with imaging functions such as fluorescence/photoacoustic/MR imaging, highly specific, robust detection of tumors can be realized for precise treatment. Owing to these excellent properties of nano-agents, they are worthy candidates for constructing tumor specific theranostic probes.

Through EPR effect, the nano-agents are able to passively target the tumor cells, thus showing a higher concentration at the tumor site (Fang et al., 2011; Lammers et al., 2012). Under irradiation, the temperature in the tumor site increases much more significantly in comparison with normal tissues, which realize the selective treatment of the tumor. For example, Li and coworkers prepared subminiature magnetic CuFeSe_2_ ternary nanocrystals, which have a wide near infrared absorption and high photothermal conversion efficiency of 500–1,100 nm (82%). These characteristics make them ideal nanotherapeutics for tumor photothermal therapy guided by multimodal imaging (such as PAI, MRI, and CT). Animal experiments show that the invasive metastasis of tumor cells can be significantly inhibited after 30 days of treatment, which proves that the imaging guided PTT of tumors assisted by CuFeSe_2_ nanocrystals has good efficacy (Jiang et al., 2017). Similarly, Zeng and coworkers prepared a kind of mixed gold nanostructure for optical/PA imaging, drug-controlled release, and PTT of tumors. The epidermal growth factor receptor (EGFR) inhibitor EB was loaded with this gold nanostructure to obtain the nano-drug EA-AB. Upon entering the cell, EB can be released via lysosomal protease and partial acid pH, meanwhile restoring the fluorescence imaging performance of AuCluster. The irradiation of NIR light will further promote the release of drugs and produce PTT effect, achieving significant inhibition of tumor growth. In addition, the biodistribution and metabolic process of nanostructures can be successfully screened by the whole-body and 3D MSOT imaging (Zhan et al., 2019). These above studies display the high efficiency of theranostic probes with NIR absorption to fight against tumors. To further improve the specificity of treatment for tumors, Shi and his coworkers developed a hydrogen sulfide (H_2_S) responsive nanoplatinum probe (Nano-PT) for NIR-II fluorescence-guided PTT of colorectal cancer (CRC). The over-expressed H_2_S in colon cancer can activate the Nano-PT to serve as a photothermal agent with high photothermal conversion efficiency, which largely improves the specificity of theranostics (Shi et al., 2018).

### Photodynamic Nanoprobes for Tumor Theranostics

Similar to photothermal therapy, photodynamic therapy (PDT), as another non-invasive and selective physical therapy, has been widely used for tumor treatment in clinic. Based on the employment of photosensitizers (PSs), which can be activated by light to generate ROS such as singlet-oxygen, PDT can destroy the tumor cells, especially mitochondria and nucleic acid (Chan et al., 2018). However, tumor cells can adapt to ROS attack by launching DNA damage repair to mitigate the damages, which could greatly decrease the therapeutic efficiency of current oxidation therapy (Juarranz et al., 2008). To amplify the tumor killing ability of PDT, Zhang’s group proposed an enhanced oxidative damage strategy for PDT, which was focused not only on the increase of ROS production but also on the simultaneous inhibition of the activity of MTH1 enzyme with DNA protection ability. Specifically, the actionable nano-system, which is a stimuli-responsive nano-system based on mesoporous silica-coated Prussian blue (PMPT) for enhanced oxidative stress of the tumor, was constructed by relieving the hypoxia-resistant situation, elevating ROS generation of PDT, and inhibiting the MTH1-regulated DNA damage repair pathway. Such PMPT could decompose the H_2_O_2_ in the tumor area to provide sufficient oxygen as the reactant of PDT. Under light irradiation, photodynamic reaction produced sufficient ROS, while the DNA repairing process can be inhibited by acid-responsive released TH287, magnifying the oxidative damage of tumor cells. In addition, the accumulation process of PMPT can be visualized by fluorescence and PA imaging, which guided the orientation of light and ensured the PDT efficiency (Hu J.-J. et al., 2019). In another work, Liu’s group prepared a kind of hollow CaCO_3_-PDA composite nanoparticle as a multi-functional treatment and diagnosis nanoplatform. The nanoparticles have high sensitivity to pH and can be rapidly degraded in the acidic microenvironment of the tumor. Therefore, the photoactivity of the loaded dihydroporphyrin E6 (CE6) photosensitizer, which was quenched by PDA in its original state, can be activated in tumor, showing recoverable fluorescence and more ROS production. In addition, due to the stable coordination between metal ions and PDA, nanoparticles can be combined with various types of metal ions, which endow them with multimodal imaging ability to guide the PDT therapy (Dong et al., 2018).

**FIGURE 7:**
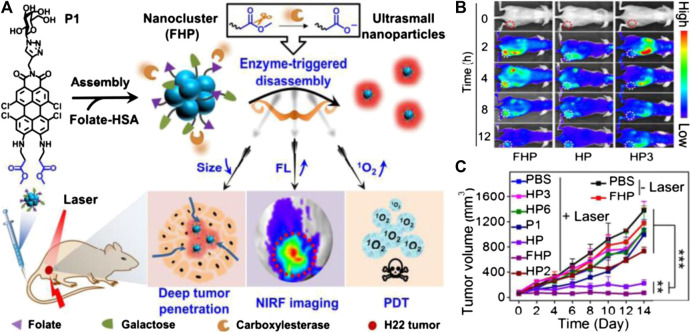
**(A)** Schematic illustrations of FHP nanocluster fabrication (assembly of P1 and folate-modified human serum albumin (HSA)), enzyme-triggered disassembly of FHP with size shrinkage for deep tumor penetration, and activatable photoactivity for near-infrared fluorescence (NIRF) imaging-guided deep PDT. **(B)** Real-time monitoring of fluorescence images *in vivo* of tumor-bearing mice after intravenous injection of FHP, HP, and HP3 (3.75 mg kg^−1^ per injection). **(C)** Tumor growth curves of H22 tumor-bearing mice treated with various agents. Reproduced with permission from Tang et al. (2019). Copyright 2020 Wiley-VCH Verlag GmbH & Co. KgaA.

At present, the most commonly used PSs in clinic are porphyrin and its derivatives, of which the excitation bands usually range from UV to visible wavelength. However, the light in such a band can be largely absorbed by tissues and scatter to lose accuracy, thereby reducing the PDT therapeutic effects in deep tumors (Rajora et al., 2017). Nano-based photosensitizers can overcome this limitation through sophisticated design. Gao’s group chemically conjugated the protoporphyrin IX molecules with jeffamines molecules to improve the hydrophilicity and biocompatibility of porphyrin, and the obtained water-soluble porphyrin-jeffamine (PJ) was further modified on the surface of PEGylated upconversion nanoparticles (UCNPs) via “click” reaction, which can construct the FRET couple between UCNP and PJs. As a result, the probes can be successfully photosensitized to generate ROS under the irradiation of 980 nm NIR light, which paves a new way to enhance the photoactivation efficiency of tumors located in deep tissues (Sun et al., 2019). Zhang and coworkers prepared an UCNP with the core-shell structure of NaErF_4_:Yb/Tm@NaYF_4_:Yb@NaNdF_4_:Yb that can be excited by two different near infrared light (NIR) at 808 and 980 nm. Through precise control of the energy transfer process, the red emission light under 980 nm excitation can trigger PDT, while the green emission under 808 nm light excitation can used for diagnosis to monitor the treatment. Using these nanoparticles as PTT agents, the tissue penetration depth has been significantly improved (Tang et al., 2019). In addition to adjusting the excitation band, the poor active penetration of the photosensitizer can be further enhanced by a well-designed responsive strategy of nanoprobes. Yi and coworkers reported a smart perylene monoimide-based nanocluster, which can be triggered by enzymes in the microenvironment to disassemble and penetrate in the deeper area of the tumor, realizing deep PDT (Figure 7). Specifically, tetrachloroperylene monoimide (P1) with the carboxylesterase (CE)-responsive ability can be further fabricated with folate-decorated albumins to construct nanostructure (FHP) with an approximate size of 100 nm. After arriving at the tumor area, P1 will be hydrolyzed by the overexpressed CE, leading to the disassembly of FHP to form ultrasmall nanoparticles of only ∼10 nm, which significantly increased their penetration depth in tumor tissue. Furthermore, during enzyme-triggered disassembly of FHP, the fluorescence intensity of this nanostructure can be largely enhanced with the elevation of ROS production, enabling the self-enhanced *in situ* NIR optical imaging as well as enhanced PDT. As a result, FHP exhibit remarkable tumor suppression ability *in vivo* with satisfied biosafety through precise imaging-guided, enzyme-triggered PDT with deep penetration (Cai Y. et al., 2020).

Although PDT is a promising strategy against tumors, the PSs will be inevitably distributed in normal tissues, especially in the skin, which may induce phototoxicity when the body is exposed to light. To address this problem, Li and Wang jointly developed an AIE type of PSs, which were loaded into liposomes to improve the stability to construct AIE-PS@liposomes. The AIE-PSs did not exhibit photosensitivity when entrapped in liposomes. After entering tumor regions, the AIE-PSs will be released from liposomes and immediately aggregate in a targeted area, which can trigger the photosensitivity of AIE-PSs at a turn-on state and induce cytotoxicity. Therefore, the AIE-PS@liposomes can significantly minimize the phototoxicity of PSs to normal tissues and organs, thereby avoiding the side effects (Yang et al., 2019).

### Magnetic Hyperthermia Nanoprobes for Tumor Theranostics

Magnetic hyperthermia (MHT) has been proposed as an alternative therapy for cancer treatment, as it can kill tumor cells with heat produced by magnetothermal agents under a strong alternating magnetic field (AMF). Unlike the aforementioned commonly developed PTT, which heats up tumors through light irradiation, MHT is able to treat deeper tumors in bodies owing to the excellent tissue penetration ability of AMF. It may also stimulate the immune function of the body itself (Mai et al., 2019), which significantly improves the therapeutic effect of tumors.

Although MHT has been shown to be an extremely powerful anti-cancer approach, the potential of this therapy is still hindered by a number of limitations, such as the non-homogeneous distribution of temperature over the tumor tissue, insufficient selectivity, and unsatisfactory patient compliance. Currently, new and high-efficiency magnetic nanomaterials, which possess not only excellent tumor targeting and penetration ability to identify the tumor tissues, realizing the selectively heated of the tumor area only under the AMF, but also possess high saturation magnetization to boost magnetothermal conversion efficiency, are being developed, promoting the application of this therapy in tumor treatment (Yavuz et al., 2006; Lacroix et al., 2010; Yoo et al., 2011; Cheng et al., 2019).

Superparamagnetic iron oxide nanoparticles, with satisfied saturation magnetization and biocompatibility, are widely employed as materials in MHT agents. Usually, these agents can also be employed as MRI or magnetic particle imaging (MPI) contrast agents to provide high resolution tumor images to monitor the process of the treatment. For example, Tian and Liang jointly developed a nano-system for active targeting tumor MHT with dual-modalities MRI/MPI capabilities. In order to improve the uniformity of its delivery, 18 nm iron oxide nanoparticles were modified by tumor targeting peptide CREKA to target tumors. With the improvement of MRI/MPI imaging, the targeting agent could significantly improve the uniformity of nanomaterials’ distribution within the tumor tissues. Through this property, MHT efficiency of tumor can be significantly improved (Du et al., 2019). Similarly, Wang and coworkers prepared ultrasmall superparamagnetic Fe_3_O_4_ nanoparticles (ES-MIONs) with a size of 3.5 nm, which can serve as a *T*_1_ contrast agent of MRI. After entering into the tumor tissue, these nanoparticles can be triggered to assemble into large-scale nanoclusters, which will largely enhance the *T*_2_ signal in MRI and simultaneously realize the MHT (Wang et al., 2017).

In another work, Fan’s group successfully prepared Uniform wüstite Fe_0.6_Mn_0.4_O nanoflowers as an innovative theranostic agent for dual-modal MRI sensitivity. After the medication of the Fe_0.6_Mn_0.4_O nanoflowers, the orthotopic glioma in a mouse model can be clearly delineated in both *T*_1_-and *T*_2_-weighted MRI. In addition, the Fe_0.6_Mn_0.4_O nanoflowers can also be employed as HMT agents, which can successfully cause the apoptosis of MCF-7 breast cancer cells and remarkably shrink the tumor with negligible side effects (Liu et al., 2016). These results have confirmed that this nano-agent could be a satisfactory magnetic theranostic platform with the capabilities of *T*_1_-*T*_2_ dual-modalities MRI for tumor imaging and MHT for tumor treatment.

However, the magnetothermal conversion efficiency determined by the specific absorption rate (SAR) of iron oxide nanoparticles is still not very high, owing to the inadequate inherent saturation magnetization (Ms) of approximately 60 emu/g. Therefore, the high local concentrations, together with high-power AMF, is required for effective MHT ablation of tumors, which is usually difficult to achieve. To address this problem, nanoparticles using pure iron or its alloys have been employed as MHT agents, as the Ms of these particles are much higher than that of iron oxide (Chao et al., 2019). For instance, very recently, Rao and Dai developed graphitic carbon-coated FeCo nanoparticles (FeCo@C), which exhibited high saturation magnetization of the FeCo core as well as strong NIR absorbance of the carbon shell, and showed a strong enhancement of MPI, high *r*_2_ MRI relaxivity, together with NIR-I and NIR-II PA imaging capability (Song et al., 2020). Such probes with magnetothermal and photothermal properties can therefore be used for tumor ablation in mice, and their high optical absorbance in a band NIR region spectral range makes them suitable as tracers for PAI.

### Nanoparticle-Enhanced Radiotherapy for Tumor Theranostics

External beam-based cancer radiotherapy is a commonly used cancer treatment strategy that has been extensively used in clinic to treat 65–75% of primary solid tumors at different stages, and employs local ionizing radiation beams, such as high-energy X-ray, γ-ray, or electron beams, to kill cancer cells (Barnett et al., 2009; Hainfeld et al., 2013; Robert et al., 2011). However, as a type of local treatment, conventional radiotherapy is not able to kill distantly spreading tumors and thus is ineffective to control tumor metastases. Unlike external beam radiotherapy, another kind of radiotherapy, which relies on the tumor-targeted systematic delivery of radioactive substances, known as radionuclide therapy, and has displayed excellent capability for the treatment of spreading tumors and lymphomas (Eroglu and Ribas, 2016). In recent years, nano-agents have been widely studied as tumor-targeting carriers of radionuclide owing to their plentitude of labeling sites.

For example, Herth and coworkers exploited a targeting agent based on polymers that can be used for pre-targeted imaging and can divide the tumor from the imaging step timely to minimize side effects during nuclear imaging and targeted radionuclide therapy. Specifically, *trans*-cyclooctene (TCO) is employed to modify the polypeptide-graftpolypeptoid polymers (PeptoBrushes) to form the targeting moiety, while the ^111^In-labeled 1,2,4,5-tetrazine derivative serves as an imaging agent to connect with TCO-modified PeptoBrushes. The sufficient contrast of the tumor can be observed 2 h post-injection of the tetrazine imaging agent, which further increases to 22 h to clearly depict the morphology of CT-26 tumors in mouse models (Steen et al., 2020). Such results demonstrate that PeptoBrushes have great potential to serve as a pre-targeting agent. In another work, Quinn and coworkers developed Lutetium-177 (^177^Lu) radiolabeled ultrasmall fluorescent silica nanoparticles (C′ dots) for effective tumor radiotherapy. Such PEGylated C′ dots with alpha melanocyte stimulating hormone (αMSH) cyclic peptide analogs on their surface can successfully recognize the melanoma tumor cells which over-expressed melanocortin-1 receptor (MC1-R). With the radiochemical stability, biological activity, and high affinity cellular binding properties of ^177^Lu-DOTA-αMSH-PEG-C′ dots, efficient tumor uptake and safe metabolic behavior were revealed through SPECT imaging in mice melanoma models. Such C′ dots successfully prolonged the survival period of mice with satisfactory biosafety, paving a new road for nuclear imaging and targeted radionuclide therapy (Zhang et al., 2020).

**FIGURE 8:**
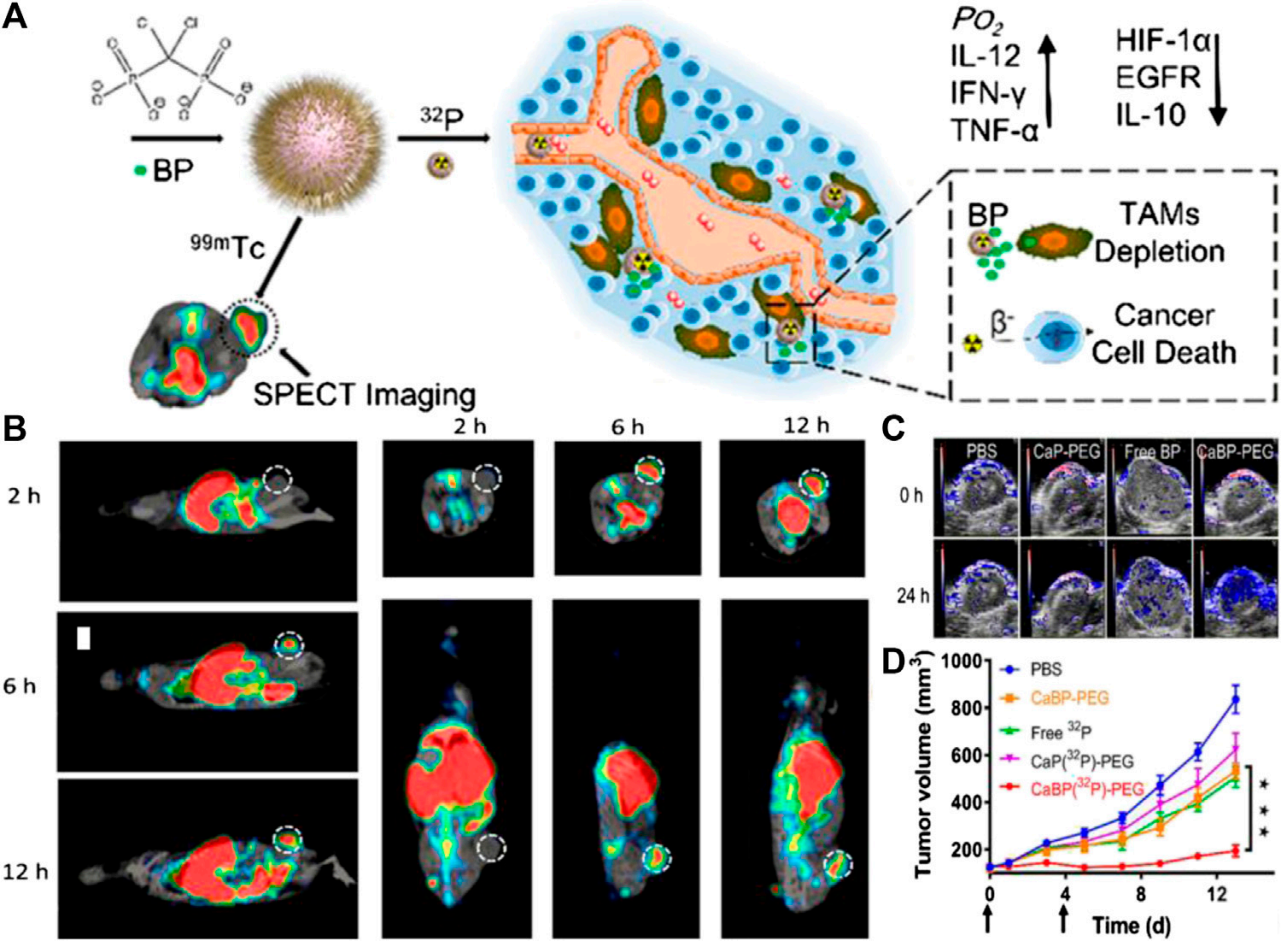
**(A)** Calcium bisphosphonate (CaBP-PEG) nanoparticles for chelator-free radiolabeling chemistry, effective *in vivo* depletion of TAMs, and imaging-guided enhanced cancerradioisotope therapy (RIT). **(B)** SPECT images of mice at 2, 6, and 12 h after intravenous injection of CaBP(^99m^Tc)-PEG nanoparticles. Tumors of mice are highlighted by the dotted circles. **(C)** Photoacoustic imaging showing tumor saturated O_2_ levels of mice after i.v. injection of PBS, CaP-PEG, free BP, or CaBP-PEG nanoparticles. **(D)** Tumor volume curves of mice with various treatments. Doses for each injection: 100 μCi of 32P, 200 μg of BP. The mice were treated twice at day 0 and 4 (black arrows). Reproduced with permission from Chen J. et al. (2019). Copyright 2018 American Chemical Society.

Radiotherapy efficacy is also severely impaired by the hypoxia of the tumor microenvironment. Recently, a variety of approaches employing nano-agents with unique physiochemical properties have been demonstrated to efficiently alleviate the hypoxic state of tumors and thus significantly promote radiotherapy. Chen and coworkers developed a biocompatible hybrid protein nanoreactor (HSA-CAT NRs) based on human serum albumin (HSA) and catalase (CAT) molecules through glutaraldehyde-mediated crosslinking, which can be further labeled with therapeutic ^131^I. Such HSA-CAT NRs can well protect the stability of enzymes, and thus can efficiently catalyze the decomposition of H_2_O_2_. HSA-CAT NRs can also accumulate with the tumor area successfully, which can be visualized under the fluorescence imaging system and gamma camera after being intravenously injected into tumor-bearing mice. More importantly, HSA-CAT NRs can significantly attenuate tumor hypoxia by decomposing endogenous H_2_O_2_ in a tumor microenvironment to produce oxygen, and thereby largely increase the therapeutic efficacy of radionuclide ^131^I (Chen J. et al., 2019). In another work, Tian and coworkers reported a biocompatible and biodegradable nanoplatform based on calcium bisphosphonate (CaBP-PEG) nanoparticles for chelator-free radiolabeling chemistry, effective *in vivo* depletion of TAMs, and imaging-guided enhanced cancer radioisotope therapy (Figure 8). After administration, CaBP(^99m^Tc)-PEG nanoparticles can actively find the tumor through the tumor homing effect, as was clearly confirmed by SPECT imaging. Due to the inhibition effect of clinical drugs to TAMs, the immunosuppression of the tumor microenvironment will be relieved. This remodeling of the microenvironment creates a favorable condition for cancer radioisotope therapy through using CaBP(^32^P)-PEG as the radio-therapeutic agent, which displayed an appropriate synergistic therapeutic effect in suppression of the tumor growth (Tian L. et al., 2018).

## Summary and Outlook

In this review, recently developed target-triggered tumor theranostic nanoprobes were summarized, which were successfully associated with various imaging modalities with distinct treatment approaches including improved chemotherapy, chemodynamic therapy, gene therapy, immunotherapy, and physical therapy. Owing to the integration of both diagnosis and treatment of cancer, these nanoprobes are obviously superior to the conventional probes with only one property, and possess powerful abilities to not only monitor and trace *in vivo* behavior themselves, but also give immediate feedback on the treatment outcome without time consumption, thereby evaluating the prognosis in real-time. This approach is helpful to customize specific treatment strategies for different patients as early as possible through a deeper understanding of the interaction between nanomedicine and tumors, and enables the establishment of patient-specific “personalized medicine” under the precision medicine plan in the near future.

Although the clinical translation of these promising multi-functional nanomaterials is highly expected, it remains challenging. The major challenges in the road map of translation include large-scale production and modification of a given nanoparticle with high reproducibility, and evaluation of safe pharmacology. As regards the production of nanoprobes on an industrial scale, the performance and pharmacokinetic behaviors of nanoprobes from different research labs, or even from different batches prepared by the same research lab, might be highly different due to the tiny variations in multiple properties. Therefore, the large-scale production of high-quality nanoprobes is the primary factor for the clinical translation of nanoprobes. Apart from that, the practical biomedical application of a newly designed theranostic nanoprobe not only depends on the imaging effect and therapeutic efficacy, but is also largely determined by its biosafety profile. In this regard, the absorption, distribution, metabolism, and excretion of nanoprobes must be studied carefully, especially for the intravenous administrated nano-formulations, which can be easily captured by the reticuloendothelial system and be retained long-term in the body. However, on the other hand, owing to the myriad variations of these sophisticated nanoprobes, the biosafety assessment becomes much more difficult compared with conventional anti-cancer drugs (Fadeel et al., 2018). Therefore, it is also very urgent to establish a decent evaluation system for these nanomedicines.

Despite the biosafety concerns and difficulties in toxicity assessment, target-triggered tumor theranostic nanoprobes, with excellent tumor diagnosis and treatment properties, will offer more viable treatment options for cancer patients in the foreseeable future.

## References

[B1] Agostinis P. Berg K. Cengel K. A. Foster T. H. Girotti A. W. Gollnick S. O. (2011). Photodynamic therapy of cancer: an update. CA Cancer J. Clin. 61, 250–281. 10.3322/caac.20114 21617154 PMC3209659

[B2] Alex S. M. Sharma C. P. (2013). Nanomedicine for gene therapy. Drug Deliv. Transl. Res. 3, 437–445. 10.1007/s13346-012-0120-0 25788352

[B3] Anh-Nguyen T. Tiberius B. Pliquett U. Urban G. A. (2016). An impedance biosensor for monitoring cancer cell attachment, spreading and drug-induced apoptosis. Sensors Actuators A: Phys. 241, 231–237. 10.1016/j.sna.2016.02.035

[B4] Bakos O. Lawson C. Rouleau S. Tai L.-H. (2018). Combining surgery and immunotherapy: turning an immunosuppressive effect into a therapeutic opportunity. J. Immunotherapy Cancer 6, 86. 10.1186/s40425-018-0398-7 PMC6122574 30176921

[B5] Barenholz Y. (2012). Doxil - the first FDA-approved nano-drug: lessons learned. J. Controlled Release 160, 117–134. 10.1016/j.jconrel.2012.03.020 22484195

[B6] Barnett G. C. West C. M. L. Dunning A. M. Elliott R. M. Coles C. E. Pharoah P. D. P. (2009). Normal tissue reactions to radiotherapy: towards tailoring treatment dose by genotype. Nat. Rev. Cancer 9, 134–142. 10.1038/nrc2587 19148183 PMC2670578

[B7] Binnewies M. Roberts E. W. Kersten K. Chan V. Fearon D. F. Merad M. (2018). Understanding the tumor immune microenvironment (TIME) for effective therapy. Nat. Med. 24, 541–550. 10.1038/s41591-018-0014-x 29686425 PMC5998822

[B8] Bobo D. Robinson K. J. Islam J. Thurecht K. J. Corrie S. R. (2016). Nanoparticle-based medicines: a review of FDA-approved materials and clinical trials to date. Pharm. Res. 33, 2373–2387. 10.1007/s11095-016-1958-5 27299311

[B9] Bol K. F. Schreibelt G. Gerritsen W. R. De Vries I. J. M. Figdor C. G. (2016). Dendritic cell-based immunotherapy: state of the art and beyond. Clin. Cancer Res. 22, 1897–1906. 10.1158/1078-0432.CCR-15-1399 27084743

[B10] Boumahdi S. De Sauvage F. J. (2020). The great escape: tumour cell plasticity in resistance to targeted therapy. Nat. Rev. Drug Discov. 19, 39–56. 10.1038/s41573-019-0044-1 31601994

[B11] Cai H. Dai X. Wang X. Tan P. Gu L. Luo Q. (2020). A nanostrategy for efficient imaging‐guided antitumor therapy through a stimuli‐responsive branched polymeric prodrug. Adv. Sci. 7, 1903243. 10.1002/advs.201903243 PMC7080516 32195104

[B12] Cai X. Xie Z. Ding B. Shao S. Liang S. Pang M. (2019). Monodispersed copper(I)‐Based nano metal-organic framework as a biodegradable drug carrier with enhanced photodynamic therapy efficacy. Adv. Sci. 6, 1900848. 10.1002/advs.201900848 PMC6685469 31406677

[B13] Cai Y. Ni D. Cheng W. Ji C. Wang Y. Müllen K. (2020). Enzyme‐triggered disassembly of perylene monoimide‐based nanoclusters for activatable and deep photodynamic therapy. Angew. Chem. Int. Ed., 59, 14014. 10.1002/anie.202001107 32363672

[B14] Chan M.-H. Pan Y.-T. Chan Y.-C. Hsiao M. Chen C.-H. Sun L. (2018). Nanobubble-embedded inorganic 808 nm excited upconversion nanocomposites for tumor multiple imaging and treatment. Chem. Sci. 9, 3141–3151. 10.1039/c8sc00108a 29732096 PMC5916109

[B15] Chang H. C. Zou Z. Z. Wang Q. H. Li J. Jin H. Yin Q. X. (2020). Targeting and specific activation of antigen‐presenting cells by endogenous antigen‐loaded nanoparticles elicits tumor‐specific immunity. Adv. Sci. 7, 1900069. 10.1002/advs.201900069 PMC6947714 31921548

[B16] Chao Y. Chen G. Liang C. Xu J. Dong Z. Han X. (2019). Iron nanoparticles for low-power local magnetic hyperthermia in combination with immune checkpoint blockade for systemic antitumor therapy. Nano Lett. 19, 4287–4296. 10.1021/acs.nanolett.9b00579 31132270

[B17] Chen H. Gu Z. An H. Chen C. Chen J. Cui R. (2018). Precise nanomedicine for intelligent therapy of cancer. Sci. China Chem. 61, 1503–1552. 10.1007/s11426-018-9397-5

[B18] Chen J. Liang C. Song X. Yi X. Yang K. Feng L. (2019). Hybrid protein nano‐reactors enable simultaneous increments of tumor oxygenation and iodine‐131 delivery for enhanced radionuclide therapy. Small 15, e1903628. 10.1002/smll.201903628 31577387

[B19] Chen Q. Wang C. Zhang X. Chen G. Hu Q. Li H. (2019). *In situ* sprayed bioresponsive immunotherapeutic gel for post-surgical cancer treatment. Nat. Nanotech. 14, 89–97. 10.1038/s41565-018-0319-4 30531990

[B20] Cheng X. Sun R. Xia H. Ding J. Yin L. Chai Z. (2019). Light-triggered crosslinking of gold nanoparticles for remarkably improved radiation therapy and computed tomography imaging of tumors. Nanomedicine 14, 2941–2955. 10.2217/nnm-2019-0015 31755353

[B21] Chiang C.-S. Lin Y.-J. Lee R. Lai Y.-H. Cheng H.-W. Hsieh C.-H. (2018). Combination of fucoidan-based magnetic nanoparticles and immunomodulators enhances tumour-localized immunotherapy. Nat. Nanotech. 13, 746–754. 10.1038/s41565-018-0146-7 29760523

[B22] De Jong W. H. Hagens W. I. Krystek P. Burger M. C. Sips A. J. A. M. Geertsma R. E. (2008). Particle size-dependent organ distribution of gold nanoparticles after intravenous administration. Biomaterials 29, 1912–1919. 10.1016/j.biomaterials.2007.12.037 18242692

[B23] Dixon S. J. Lemberg K. M. Lamprecht M. R. Skouta R. Zaitsev E. M. Gleason C. E. (2012). Ferroptosis: an iron-dependent form of nonapoptotic cell death. Cell 149, 1060–1072. 10.1016/j.cell.2012.03.042 22632970 PMC3367386

[B24] Dong Z. Feng L. Hao Y. Chen M. Gao M. Chao Y. (2018). Synthesis of hollow biomineralized CaCO3-polydopamine nanoparticles for multimodal imaging-guided cancer photodynamic therapy with reduced skin photosensitivity. J. Am. Chem. Soc. 140, 2165–2178. 10.1021/jacs.7b11036 29376345

[B25] Dougherty T. J. Gomer C. J. Henderson B. W. Jori G. Kessel D. Korbelik M. (1998). Photodynamic therapy. JNCI J. Natl. Cancer Inst. 90, 889–905. 10.1093/jnci/90.12.889 9637138 PMC4592754

[B27] Du K. Liu Q. Liu M. Lv R. He N. Wang Z. (2020). Encapsulation of glucose oxidase in Fe(III)/tannic acid nanocomposites for effective tumor ablation via Fenton reaction. Nanotechnology 31, 015101. 10.1088/1361-6528/ab44f9 31530753

[B26] Du Y. Liu X. Liang Q. Liang X.-J. Tian J. (2019). Optimization and design of magnetic ferrite nanoparticles with uniform tumor distribution for highly sensitive MRI/MPI performance and improved magnetic hyperthermia therapy. Nano Lett. 19, 3618–3626. 10.1021/acs.nanolett.9b00630 31074627

[B28] Duro-Castano A. Gallon E. Decker C. Vicent M. J. (2017). Modulating angiogenesis with integrin-targeted nanomedicines. Adv. Drug Deliv. Rev. 119, 101–119. 10.1016/j.addr.2017.05.008 28502767

[B29] Eroglu Z. Ribas A. (2016). Combination therapy with BRAF and MEK inhibitors for melanoma: latest evidence and place in therapy. Ther. Adv. Med. Oncol. 8, 48–56. 10.1177/1758834015616934 26753005 PMC4699264

[B30] Fadeel B. Farcal L. Hardy B. Vázquez-Campos S. Hristozov D. Marcomini A. (2018). Advanced tools for the safety assessment of nanomaterials. Nat. Nanotech. 13, 537–543. 10.1038/s41565-018-0185-0 29980781

[B31] Fang J. Nakamura H. Maeda H. (2011). The EPR effect: unique features of tumor blood vessels for drug delivery, factors involved, and limitations and augmentation of the effect. Adv. Drug Deliv. Rev. 63, 136–151. 10.1016/j.addr.2010.04.009 20441782

[B32] Fenton O. S. Kauffman K. J. Mcclellan R. L. Kaczmarek J. C. Zeng M. D. Andresen J. L. (2018). Customizable lipid nanoparticle materials for the delivery of siRNAs and mRNAs. Angew. Chem. Int. Ed. 57, 13582–13586. 10.1002/anie.201809056 PMC7548314 30112821

[B33] Fridman W. H. Pagès F. Sautès-Fridman C. Galon J. (2012). The immune contexture in human tumours: impact on clinical outcome. Nat. Rev. Cancer 12, 298–306. 10.1038/nrc3245 22419253

[B34] Fu J. Li K. Zhang W. Wan C. Zhang J. Jiang P. (2020). Large-scale public data reuse to model immunotherapy response and resistance. Genome Med. 12. 10.1186/s13073-020-0721-z PMC7045518 32102694

[B35] Gajewski T. F. Meng Y. Blank C. Brown I. Kacha A. Kline J. (2006). Immune resistance orchestrated by the tumor microenvironment. Immunol. Rev. 213, 131–145. 10.1111/j.1600-065X.2006.00442.x 16972901

[B36] Gao Z. Hou Y. Zeng J. Chen L. Liu C. Yang W. (2017). Tumor microenvironment-triggered aggregation of antiphagocytosis 99m Tc-labeled Fe3 O4 nanoprobes for enhanced tumor imaging in vivo. Adv. Mater. 29, 1701095. 10.1002/adma.201701095 28402594

[B37] Grahnert A. Grahnert A. Klein C. Schilling E. Wehrhahn J. Hauschildt S. (2011). Review: NAD+: a modulator of immune functions. Innate Immun. 17, 212–233. 10.1177/1753425910361989 20388721

[B38] Grippin A. J. Wummer B. Wildes T. Dyson K. Trivedi V. Yang C. (2019). Dendritic cell-activating magnetic nanoparticles enable early prediction of antitumor response with magnetic resonance imaging. ACS Nano 13, 13884–13898. 10.1021/acsnano.9b05037 31730332 PMC7182054

[B39] Gu Z. Zhu S. Yan L. Zhao F. Zhao Y. (2019). Graphene-based smart platforms for combined cancer therapy. Adv. Mater. 31, 1800662. 10.1002/adma.201800662 30039878

[B40] Guo S. Huang L. (2014). Nanoparticles containing insoluble drug for cancer therapy. Biotechnol. Adv. 32, 778–788. 10.1016/j.biotechadv.2013.10.002 24113214 PMC3980181

[B41] Gupta P. Lakes A. Dziubla T. (2016). A free radical primer. Oxidative Stress Biomater. 2016, 1–33. 10.1016/b978-0-12-803269-5.00001-2

[B42] Hainfeld J. F. Smilowitz H. M. O’Connor M. J. Dilmanian F. A. Slatkin D. N. (2013). Gold nanoparticle imaging and radiotherapy of brain tumors in mice. Nanomedicine 8, 1601–1609. 10.2217/nnm.12.165 23265347 PMC3657324

[B43] Han B. Mao F.-Y. Zhao Y.-L. Lv Y.-P. Teng Y.-S. Duan M. (2018). Altered NKp30, NKp46, NKG2D, and DNAM-1 expression on circulating NK cells is associated with tumor progression in human gastric cancer. J. Immunol. Res. 2018, 1. 10.1155/2018/6248590 PMC6140275 30255106

[B44] Han H. Hou Y. Chen X. Zhang P. Kang M. Jin Q. (2020). Metformin-induced stromal depletion to enhance the penetration of gemcitabine-loaded magnetic nanoparticles for pancreatic cancer targeted therapy. J. Am. Chem. Soc. 142, 4944–4954. 10.1021/jacs.0c00650 32069041

[B45] Han K. Chen S. Chen W.-H. Lei Q. Liu Y. Zhuo R.-X. (2013). Synergistic gene and drug tumor therapy using a chimeric peptide. Biomaterials 34, 4680–4689. 10.1016/j.biomaterials.2013.03.010 23537665

[B46] Hattermann K. Sebens S. Helm O. Schmitt A. D. Mentlein R. Mehdorn H. M. (2014). Chemokine expression profile of freshly isolated human glioblastoma-associated macrophages/microglia. Oncol. Rep. 32, 270–276. 10.3892/or.2014.3214 24859792

[B47] He X. Yin F. Wang D. Xiong L.-H. Kwok R. T. K. Gao P. F. (2019). AIE featured inorganic-organic Core@Shell nanoparticles for high-efficiency siRNA delivery and real-time monitoring. Nano Lett. 19, 2272–2279. 10.1021/acs.nanolett.8b04677 30829039

[B48] High K. A. Roncarolo M. G. (2019). Gene therapy. N. Engl. J. Med. 381, 455–464. 10.1056/NEJMra1706910 31365802

[B49] Hu J.-J. Chen Y. Li Z.-H. Peng S.-Y. Sun Y. Zhang X.-Z. (2019). Augment of oxidative damage with enhanced photodynamic process and MTH1 inhibition for tumor therapy. Nano Lett. 19, 5568–5576. 10.1021/acs.nanolett.9b02112 31262183

[B50] Hu R. Fang Y. Huo M. Yao H. Wang C. Chen Y. (2019). Ultrasmall Cu2-xS nanodots as photothermal-enhanced Fenton nanocatalysts for synergistic tumor therapy at NIR-II biowindow. Biomaterials 206, 101–114. 10.1016/j.biomaterials.2019.03.014 30927714

[B51] Huang G. Zhang K.-L. Chen S. Li S.-H. Wang L.-L. Wang L.-P. (2017). Manganese-iron layered double hydroxide: a theranostic nanoplatform with pH-responsive MRI contrast enhancement and drug release. J. Mater. Chem. B 5, 3629–3633. 10.1039/c7tb00794a 32264050

[B52] Huang L.-L. Li X. Zhang J. Zhao Q. R. Zhang M. J. Liu A.-A. (2019). MnCaCs-biomineralized oncolytic virus for bimodal imaging-guided and synergistically enhanced anticancer therapy. Nano Lett. 19, 8002–8009. 10.1021/acs.nanolett.9b03193 31626554

[B53] Huo M. Wang L. Wang Y. Chen Y. Shi J. (2019). Nanocatalytic tumor therapy by single-atom catalysts. ACS Nano 13, 2643–2653. 10.1021/acsnano.9b00457 30753056

[B54] Irvine D. J. Dane E. L. (2020). Enhancing cancer immunotherapy with nanomedicine. Nat. Rev. Immunol. 20, 321–334. 10.1038/s41577-019-0269-6 32005979 PMC7536618

[B55] Janib S. M. Moses A. S. Mackay J. A. (2010). Imaging and drug delivery using theranostic nanoparticles. Adv. Drug Deliv. Rev. 62, 1052–1063. 10.1016/j.addr.2010.08.004 20709124 PMC3769170

[B56] Jiang Q. Zhao S. Liu J. Song L. Wang Z.-G. Ding B. (2019). Rationally designed DNA-based nanocarriers. Adv. Drug Deliv. Rev. 147, 2–21. 10.1016/j.addr.2019.02.003 30769047

[B57] Jiang X. Zhang S. Ren F. Chen L. Zeng J. Zhu M. (2017). Ultrasmall magnetic CuFeSe2 ternary nanocrystals for multimodal imaging guided photothermal therapy of cancer. ACS Nano 11, 5633–5645. 10.1021/acsnano.7b01032 28525715

[B58] Jing L. Yang C. Zhang P. Zeng J. Li Z. Gao M. (2020). Nanoparticles weaponized with built‐in functions for imaging‐guided cancer therapy. View, 1, e19. 10.1002/viw2.19

[B59] Juarranz Á. Jaén P. Sanz-Rodríguez F. Cuevas J. González S. (2008). Photodynamic therapy of cancer. Basic principles and applications. Clin. Transl Oncol. 10, 148–154. 10.1007/s12094-008-0172-2 18321817

[B60] Kelkar S. S. Reineke T. M. (2011). Theranostics: combining imaging and therapy. Bioconjug. Chem. 22, 1879–1903. 10.1021/bc200151q 21830812

[B61] Kim B. Sun S. Varner J. A. Howell S. B. Ruoslahti E. Sailor M. J. (2019). Securing the payload, finding the cell, and avoiding the endosome: peptide‐targeted, fusogenic porous silicon nanoparticles for delivery of siRNA. Adv. Mater. 31, 1902952. 10.1002/adma.201902952 PMC6710136 31267590

[B62] Kim J. Lee Y. M. Kim H. Park D. Kim J. Kim W. J. (2016). Phenylboronic acid-sugar grafted polymer architecture as a dual stimuli-responsive gene carrier for targeted anti-angiogenic tumor therapy. Biomaterials 75, 102–111. 10.1016/j.biomaterials.2015.10.022 26491998

[B63] Koppers-Lalic D. Hogenboom M. M. Middeldorp J. M. Pegtel D. M. (2013). Virus-modified exosomes for targeted RNA delivery; a new approach in nanomedicine. Adv. Drug Deliv. Rev. 65, 348–356. 10.1016/j.addr.2012.07.006 22820525 PMC7103310

[B64] Kotterman M. A. Chalberg T. W. Schaffer D. V. (2015). Viral vectors for gene therapy: translational and clinical outlook. Annu. Rev. Biomed. Eng. 17, 63–89. 10.1146/annurev-bioeng-071813-104938 26643018

[B65] Kuang H. Ku S. H. Kokkoli E. (2017). The design of peptide-amphiphiles as functional ligands for liposomal anticancer drug and gene delivery. Adv. Drug Deliv. Rev. 110-111, 80–101. 10.1016/j.addr.2016.08.005 27539561

[B66] Lacroix L.-M. Ho D. Sun S. (2010). Magnetic nanoparticles as both imaging probes and therapeutic agents. Ctmc 10, 1184–1197. 10.2174/156802610791384207 20388109

[B67] Lammers T. Kiessling F. Hennink W. E. Storm G. (2012). Drug targeting to tumors: principles, pitfalls and (pre-) clinical progress. J. Controlled Release 161, 175–187. 10.1016/j.jconrel.2011.09.063 21945285

[B68] Lee J. Jeong E. J. Lee Y. K. Kim K. Kwon I. C. Lee K. Y. (2016). Optical imaging and gene therapy with neuroblastoma-targeting polymeric nanoparticles for potential theranostic applications. Small 12, 1201–1211. 10.1002/smll.201501913 26573885

[B69] Li F. Nie W. Zhang F. Lu G. Lv C. Lv Y. (2019). Engineering magnetosomes for high-performance cancer vaccination. ACS Cent. Sci. 5, 796–807. 10.1021/acscentsci.9b00060 31139716 PMC6535768

[B70] Lin L.-S. Song J. Song L. Ke K. Liu Y. Zhou Z. (2018). Simultaneous fenton-like ion delivery and glutathione depletion by MnO2 -based nanoagent to enhance chemodynamic therapy. Angew. Chem. Int. Ed. 57, 4902–4906. 10.1002/anie.201712027 29488312

[B71] Liu B. Hu F. Zhang J. Wang C. Li L. (2019). A biomimetic coordination nanoplatform for controlled encapsulation and delivery of drug-gene combinations. Angew. Chem. Int. Ed. 58, 8804–8808. 10.1002/anie.201903417 31033145

[B72] Liu D. Zhou Z. Wang X. Deng H. Sun L. Lin H. (2020). Yolk-shell nanovesicles endow glutathione-responsive concurrent drug release and T1 MRI activation for cancer theranostics. Biomaterials 244, 119979. 10.1016/j.biomaterials.2020.119979 32200104 PMC7138217

[B73] Liu H. Moynihan K. D. Zheng Y. Szeto G. L. Li A. V. Huang B. (2014). Structure-based programming of lymph-node targeting in molecular vaccines. Nature 507, 519–522. 10.1038/nature12978 24531764 PMC4069155

[B74] Liu J. Chen Q. Zhu W. Yi X. Yang Y. Dong Z. (2017). Nanoscale-coordination-polymer-shelled manganese dioxide composite nanoparticles: a multistage redox/pH/H2O2-responsive cancer theranostic nanoplatform. Adv. Funct. Mater. 27, 1605926. 10.1002/adfm.201605926

[B75] Liu J. Li H.-J. Luo Y.-L. Xu C.-F. Du X.-J. Du J.-Z. (2019). Enhanced primary tumor penetration facilitates nanoparticle draining into lymph nodes after systemic injection for tumor metastasis inhibition. ACS Nano 13, 8648–8658. 10.1021/acsnano.9b03472 31328920

[B76] Liu S. Zhang Y. Zhao X. Wang J. Di C. Zhao Y. (2019). Tumor-specific silencing of tissue factor suppresses metastasis and prevents cancer-associated hypercoagulability. Nano Lett. 19, 4721–4730. 10.1021/acs.nanolett.9b01785 31180684

[B77] Liu T. Tong L. Lv N. Ge X. Fu Q. Gao S. (2019). Two‐stage size decrease and enhanced photoacoustic performance of stimuli‐responsive polymer‐gold nanorod assembly for increased tumor penetration. Adv. Funct. Mater. 29, 1806429. 10.1002/adfm.201806429

[B78] Liu X. L. Ng C. T. Chandrasekharan P. Yang H. T. Zhao L. Y. Peng E. (2016). Synthesis of ferromagnetic Fe0.6Mn0.4O nanoflowers as a new class of magnetic theranostic platform for in vivo T1-T2Dual-mode magnetic resonance imaging and magnetic hyperthermia therapy. Adv. Health Mater. 5, 2092–2104. 10.1002/adhm.201600357 27297640

[B79] Liu X. Yan B. Li Y. Ma X. Jiao W. Shi K. (2020). Graphene oxide-grafted magnetic nanorings mediated magnetothermodynamic therapy favoring reactive oxygen species-related immune response for enhanced antitumor efficacy. ACS Nano 14, 1936–1950. 10.1021/acsnano.9b08320 31961656

[B80] Liu Y. Bhattarai P. Dai Z. Chen X. (2019a). Photothermal therapy and photoacoustic imaging via nanotheranostics in fighting cancer. Chem. Soc. Rev. 48, 2053–2108. 10.1039/c8cs00618k 30259015 PMC6437026

[B81] Liu Y. Wu J. Jin Y. Zhen W. Wang Y. Liu J. (2019b). Copper(I) phosphide nanocrystals for in situ self‐generation magnetic resonance imaging‐guided photothermal‐enhanced chemodynamic synergetic therapy resisting deep‐seated tumor. Adv. Funct. Mater. 29, 1904678. 10.1002/adfm.201904678

[B82] Liu Y. Deisseroth A. (2006). Tumor vascular targeting therapy with viral vectors. Blood 107, 3027–3033. 10.1182/blood-2005-10-4114 16373660

[B83] Lu Y. Aimetti A. A. Langer R. Gu Z. (2016). Bioresponsive materials. Nat. Rev. Mater. 2, 16075. 10.1038/natrevmats.2016.75

[B84] Lundstrom K. (2003). Latest development in viral vectors for gene therapy. Trends Biotechnol. 21, 117–122. 10.1016/s0167-7799(02)00042-2 12628368

[B85] Lv P. Liu X. Chen X. Liu C. Zhang Y. Chu C. (2019). Genetically engineered cell membrane nanovesicles for oncolytic adenovirus delivery: a versatile platform for cancer virotherapy. Nano Lett. 19, 2993–3001. 10.1021/acs.nanolett.9b00145 30964695

[B86] Ma T. Hou Y. Zeng J. Liu C. Zhang P. Jing L. (2018). Dual-ratiometric target-triggered fluorescent probe for simultaneous quantitative visualization of tumor microenvironment protease activity and pH *in vivo* . J. Am. Chem. Soc. 140, 211–218. 10.1021/jacs.7b08900 29237264

[B87] Ma T. Zhang P. Hou Y. Ning H. Wang Z. Huang J. (2018). “Smart” nanoprobes for visualization of tumor microenvironments. Adv. Health Mater. 7, 1800391. 10.1002/adhm.201800391 29999250

[B88] Mai B. T. Balakrishnan P. B. Barthel M. J. Piccardi F. Niculaes D. Marinaro F. (2019). Thermoresponsive iron oxide nanocubes for an effective clinical translation of magnetic hyperthermia and heat-mediated chemotherapy. ACS Appl. Mater. Inter. 11, 5727–5739. 10.1021/acsami.8b16226 PMC6376448 30624889

[B89] Mangraviti A. Tzeng S. Y. Kozielski K. L. Wang Y. Jin Y. Gullotti D. (2015). Polymeric nanoparticles for nonviral gene therapy extend brain tumor survival *in vivo* . ACS Nano 9, 1236–1249. 10.1021/nn504905q 25643235 PMC4342728

[B90] Mi P. Kokuryo D. Cabral H. Wu H. Terada Y. Saga T. (2016). A pH-activatable nanoparticle with signal-amplification capabilities for non-invasive imaging of tumour malignancy. Nat. Nanotech 11, 724–730. 10.1038/nnano.2016.72 27183055

[B91] Miao L. Li L. Huang Y. Delcassian D. Chahal J. Han J. (2019). Delivery of mRNA vaccines with heterocyclic lipids increases anti-tumor efficacy by STING-mediated immune cell activation. Nat. Biotechnol. 37, 1174–1185. 10.1038/s41587-019-0247-3 31570898

[B92] Milling L. Zhang Y. Irvine D. J. (2017). Delivering safer immunotherapies for cancer. Adv. Drug Deliv. Rev. 114, 79–101. 10.1016/j.addr.2017.05.011 28545888 PMC5647831

[B93] Mohme M. Riethdorf S. Pantel K. (2017). Circulating and disseminated tumour cells - mechanisms of immune surveillance and escape. Nat. Rev. Clin. Oncol. 14, 155–167. 10.1038/nrclinonc.2016.144 27644321

[B94] Mura S. Nicolas J. Couvreur P. (2013). Stimuli-responsive nanocarriers for drug delivery. Nat. Mater 12, 991–1003. 10.1038/nmat3776 24150417

[B95] Ni R. Zhou J. Hossain N. Chau Y. (2016). Virus-inspired nucleic acid delivery system: linking virus and viral mimicry. Adv. Drug Deliv. Rev. 106, 3–26. 10.1016/j.addr.2016.07.005 27473931

[B96] Nie W. Wei W. Zuo L. Lv C. Zhang F. Lu G.-H. (2019). Magnetic nanoclusters armed with responsive PD-1 antibody synergistically improved adoptive T-cell therapy for solid tumors. ACS Nano 13, 1469–1478. 10.1021/acsnano.8b07141 30763076

[B97] Omidi Y. (2011). Smart multifunctional theranostics: simultaneous diagnosis and therapy of cancer. Bioimpacts 1, 145–147. 10.5681/bi.2011.019 23678419 PMC3648960

[B98] Oun R. Moussa Y. E. Wheate N. J. (2018). The side effects of platinum-based chemotherapy drugs: a review for chemists. Dalton Trans. 47, 6645–6653. 10.1039/c8dt00838h 29632935

[B99] Ovais M. Guo M. Chen C. (2019). Tailoring nanomaterials for targeting tumor‐associated macrophages. Adv. Mater. 31, 1808303. 10.1002/adma.201808303 30883982

[B100] Ozpolat B. Sood A. K. Lopez-Berestein G. (2014). Liposomal siRNA nanocarriers for cancer therapy. Adv. Drug Deliv. Rev. 66, 110–116. 10.1016/j.addr.2013.12.008 24384374 PMC4527165

[B101] Pan Y.-B. Wang S. He X. Tang W. Wang J. Shao A. (2019). A combination of glioma *in vivo* imaging and *in vivo* drug delivery by metal-organic framework based composite nanoparticles. J. Mater. Chem. B 7, 7683–7689. 10.1039/c9tb01651a 31778139

[B102] Peters B. G. (1994). An overview of chemotherapy toxicities. Top. Hosp. Pharm. Manage. 14, 59–88. 10136204

[B103] Petersen G. H. Alzghari S. K. Chee W. Sankari S. S. La-Beck N. M. (2016). Meta-analysis of clinical and preclinical studies comparing the anticancer efficacy of liposomal versus conventional non-liposomal doxorubicin. J. Controlled Release 232, 255–264. 10.1016/j.jconrel.2016.04.028 27108612

[B104] Poon W. Zhang Y.-N. Ouyang B. Kingston B. R. Wu J. L. Y. Wilhelm S. (2019). Elimination pathways of nanoparticles. ACS nano 13, 5785–5798. 10.1021/acsnano.9b01383 30990673

[B105] Rajora M. A. Lou J. W. H. Zheng G. (2017). Advancing porphyrin's biomedical utility via supramolecular chemistry. Chem. Soc. Rev. 46, 6433–6469. 10.1039/c7cs00525c 29048439

[B106] Ren Z. Sun S. Sun R. Cui G. Hong L. Rao B. (2020). A metal-polyphenol‐coordinated nanomedicine for synergistic cascade cancer chemotherapy and chemodynamic therapy. Adv. Mater. 32, 1906024. 10.1002/adma.201906024 31834662

[B107] Rezaee R. Momtazi A. A. Monemi A. Sahebkar A. (2017). Curcumin: a potentially powerful tool to reverse cisplatin-induced toxicity. Pharmacol. Res. 117, 218–227. 10.1016/j.phrs.2016.12.037 28042086

[B108] Robert C. Thomas L. Bondarenko I. O'day S. Weber J. Garbe C. (2011). Ipilimumab plus dacarbazine for previously untreated metastatic melanoma. N. Engl. J. Med. 364, 2517–2526. 10.1056/NEJMoa1104621 21639810

[B109] Rodell C. B. Arlauckas S. P. Cuccarese M. F. Garris C. S. Li R. Ahmed M. S. (2018). TLR7/8-agonist-loaded nanoparticles promote the polarization of tumour-associated macrophages to enhance cancer immunotherapy. Nat. Biomed. Eng. 2, 578–588. 10.1038/s41551-018-0236-8 31015631 PMC6192054

[B110] Saha M. (2009). Nanomedicine: promising tiny machine for the healthcare in future-a review. Omj 24, 242–247. 10.5001/omj.2009.50 22216376 PMC3243873

[B111] Santiago-Ortiz J. L. Schaffer D. V. (2016). Adeno-associated virus (AAV) vectors in cancer gene therapy. J. Controlled Release 240, 287–301. 10.1016/j.jconrel.2016.01.001 PMC4940329 26796040

[B112] Schoenfeld J. D. Sibenaller Z. A. Mapuskar K. A. Wagner B. A. Cramer-Morales K. L. Furqan M. (2017). O_2_−and H_2_O2 -mediated disruption of Fe metabolism causes the differential susceptibility of NSCLC and GBM cancer cells to pharmacological ascorbate. Cancer cell 31, 487–500. 10.1016/j.ccell.2017.02.018 28366679 PMC5497844

[B113] Schöttler S. Landfester K. Mailänder V. (2016). Controlling the stealth effect of nanocarriers through understanding the protein corona. Angew. Chem. Int. Ed. 55, 8806–8815. 10.1002/anie.201602233 27303916

[B114] Shi B. Yan Q. Tang J. Xin K. Zhang J. Zhu Y. (2018). Hydrogen sulfide-activatable second near-infrared fluorescent nanoassemblies for targeted photothermal cancer therapy. Nano Lett. 18, 6411–6416. 10.1021/acs.nanolett.8b02767 30239208

[B115] Shin H. Na K. (2019). *In situ* vaccination with biocompatibility controllable immuno-sensitizer inducing antitumor immunity. Biomaterials 197, 32–40. 10.1016/j.biomaterials.2019.01.015 30639548

[B116] Shu Y. Pi F. Sharma A. Rajabi M. Haque F. Shu D. (2014). Stable RNA nanoparticles as potential new generation drugs for cancer therapy. Adv. Drug Deliv. Rev. 66, 74–89. 10.1016/j.addr.2013.11.006 24270010 PMC3955949

[B117] Singh A. Nandwana V. Rink J. S. Ryoo S.-R. Chen T. H. Allen S. D. (2019). Biomimetic magnetic nanostructures: a theranostic platform targeting lipid metabolism and immune response in lymphoma. ACS Nano 13, 10301–10311. 10.1021/acsnano.9b03727 31487458

[B118] Song C. Phuengkham H. Kim Y. S. Dinh V. V. Lee I. Shin I. W. (2019). Syringeable immunotherapeutic nanogel reshapes tumor microenvironment and prevents tumor metastasis and recurrence. Nat. Commun. 10, 3745. 10.1038/s41467-019-11730-8 31431623 PMC6702226

[B119] Song G. Kenney M. Chen Y.-S. Zheng X. Deng Y. Chen Z. (2020). Carbon-coated FeCo nanoparticles as sensitive magnetic-particle-imaging tracers with photothermal and magnetothermal properties. Nat. Biomed. Eng. 4, 325–334. 10.1038/s41551-019-0506-0 32015409 PMC7071985

[B120] Song Q. Zheng C. Jia J. Zhao H. Feng Q. Zhang H. (2019). A probiotic spore‐based oral autonomous nanoparticles generator for cancer therapy. Adv. Mater. 31, 1903793. 10.1002/adma.201903793 31490587

[B121] Song W. Das M. Xu Y. Si X. Zhang Y. Tang Z. (2019). Leveraging biomaterials for cancer immunotherapy: targeting pattern recognition receptors. Mater. Today Nano 5, 100029. 10.1016/j.mtnano.2019.100029

[B122] Song Y. Shi Y. Huang M. Wang W. Wang Y. Cheng J. (2019). Bioinspired engineering of a multivalent aptamer‐functionalized nanointerface to enhance the capture and release of circulating tumor cells. Angew. Chem. Int. Ed. 58, 2236–2240. 10.1002/anie.201809337 30548959

[B123] Sousa S. Brion R. Lintunen M. Kronqvist P. Sandholm J. Mönkkönen J. (2015). Human breast cancer cells educate macrophages toward the M2 activation status. Breast Cancer Res. 17, 101. 10.1186/s13058-015-0621-0 26243145 PMC4531540

[B124] Sprooten J. Ceusters J. Coosemans A. Agostinis P. De Vleeschouwer S. Zitvogel L. (2019). Trial watch: dendritic cell vaccination for cancer immunotherapy. Oncoimmunology 8, 1638212. 10.1080/2162402X.2019.1638212 PMC6791419 31646087

[B125] Stéen E. J. L. Jørgensen J. T. Johann K. Nørregaard K. Sohr B. Svatunek D. (2020). Trans-cyclooctene-Functionalized PeptoBrushes with improved reaction kinetics of the tetrazine ligation for pretargeted nuclear imaging. ACS Nano 14, 568–584. 10.1021/acsnano.9b06905 31820928 PMC7075664

[B126] Sumer B. Gao J. (2008). Theranostic nanomedicine for cancer. Nanomedicine 3, 137–140. 10.2217/17435889.3.2.137 18373419

[B127] Sun W. Liu X.-Y. Ma L.-L. Lu Z.-L. (2020). Tumor targeting gene vector for visual tracking of bcl-2 siRNA transfection and anti-tumor therapy. ACS Appl. Mater. Inter. 12, 10193–10201. 10.1021/acsami.0c00652 32045197

[B128] Sun X. Zhang P. Hou Y. Li Y. Huang X. Wang Z. (2019). Upconversion luminescence mediated photodynamic therapy through hydrophilically engineered porphyrin. Chem. Eng. Process 142, 107551. 10.1016/j.cep.2019.107551

[B129] Tang M. Zhu X. Zhang Y. Zhang Z. Zhang Z. Mei Q. (2019). Near-infrared excited orthogonal emissive upconversion nanoparticles for imaging-guided on-demand therapy. ACS Nano 13, 10405–10418. 10.1021/acsnano.9b04200 31448898

[B130] Tang Z. Liu Y. He M. Bu W. (2018). Chemodynamic therapy: tumour microenvironment‐mediated fenton and fenton‐like reactions. Angew. Chem. 131, 958–968. 10.1002/ange.201805664 30048028

[B131] Tang Z. Zhang H. Liu Y. Ni D. Zhang H. Zhang J. (2017). Antiferromagnetic pyrite as the tumor microenvironment-mediated nanoplatform for self-enhanced tumor imaging and therapy. Adv. Mater. 29, 1701683. 10.1002/adma.201701683 29094389

[B132] Tee J. K. Yip L. X. Tan E. S. Santitewagun S. Prasath A. Ke P. C. (2019). Nanoparticles' interactions with vasculature in diseases. Chem. Soc. Rev. 48, 5381–5407. 10.1039/c9cs00309f 31495856

[B133] Tian L. Yi X. Dong Z. Xu J. Liang C. Chao Y. (2018). Calcium bisphosphonate nanoparticles with chelator-free radiolabeling to deplete tumor-associated macrophages for enhanced cancer radioisotope therapy. ACS Nano 12, 11541–11551. 10.1021/acsnano.8b06699 30359515

[B134] Tian Y. Zhou M. Shi H. Gao S. Xie G. Zhu M. 2018). Integration of cell-penetrating peptides with rod-like bionanoparticles: virus-inspired gene-silencing technology. Nano Lett. 18, 5453–5460. 10.1021/acs.nanolett.8b01805 30091612

[B135] Torrente-Rodríguez R. M. Tu J. Yang Y. Min J. Wang M. Song Y. (2020). Investigation of cortisol dynamics in human sweat using a graphene-based wireless mHealth system. Matter, 2, 921. 10.1016/j.matt.2020.01.021 32266329 PMC7138219

[B136] Tsouris V. Joo M. K. Kim S. H. Kwon I. C. Won Y.-Y. (2014). Nano carriers that enable co-delivery of chemotherapy and RNAi agents for treatment of drug-resistant cancers. Biotechnol. Adv. 32, 1037–1050. 10.1016/j.biotechadv.2014.05.006 24924617

[B137] Uhel F. Azzaoui I. Grégoire M. Pangault C. Dulong J. Tadié J.-M. (2017). Early expansion of circulating granulocytic myeloid-derived suppressor cells predicts development of nosocomial infections in patients with sepsis. Am. J. Respir. Crit. Care Med. 196, 315–327. 10.1164/rccm.201606-1143OC 28146645

[B138] Vangasseri D. P. Cui Z. Chen W. Hokey D. A. Falo L. D. Huang L. (2009). Immunostimulation of dendritic cells by cationic liposomes. Mol. Membr. Biol. 23, 385–395. 10.1080/09687860600790537 17060156

[B139] Waehler R. Russell S. J. Curiel D. T. (2007). Engineering targeted viral vectors for gene therapy. Nat. Rev. Genet. 8, 573–587. 10.1038/nrg2141 17607305 PMC7097627

[B140] Wang F. Gao J. Xiao J. Du J. (2018). Dually gated polymersomes for gene delivery. Nano Lett. 18, 5562–5568. 10.1021/acs.nanolett.8b01985 30052457

[B141] Wang J. Mi P. Lin G. Wáng Y. X. J. Liu G. Chen X. (2016). Imaging-guided delivery of RNAi for anticancer treatment. Adv. Drug Deliv. Rev. 104, 44–60. 10.1016/j.addr.2016.01.008 26805788 PMC5226392

[B142] Wang L. Huang J. Chen H. Wu H. Xu Y. Li Y. (2017). Exerting enhanced permeability and retention effect driven delivery by ultrafine iron oxide nanoparticles with T1-T2 switchable magnetic resonance imaging contrast. ACS Nano 11, 4582–4592. 10.1021/acsnano.7b00038 28426929 PMC5701890

[B143] Wang S. Wang Z. Yu G. Zhou Z. Jacobson O. Liu Y. (2019). Tumor-specific drug release and reactive oxygen species generation for cancer chemo/chemodynamic combination therapy. Adv. Sci. 6, 1801986. 10.1002/advs.201801986 PMC6402284 30886808

[B144] Wang T. Zhang H. Liu H. Yuan Q. Ren F. Han Y. (2019). Boosting H 2 O 2 ‐guided chemodynamic therapy of cancer by enhancing reaction kinetics through versatile biomimetic fenton nanocatalysts and the second near‐infrared light irradiation. Adv. Funct. Mater. 30, 1906128. 10.1002/adfm.201906128

[B145] Wang Y. Yu C. (2020). Emerging concepts of nanobiotechnology in mRNA delivery. Angew. Chem. Int. Ed., 59, 23374. 10.1002/anie.202003545 32400110

[B146] Wang Z. Xue X. Lu H. He Y. Lu Z. Chen Z. (2020). Two-way magnetic resonance tuning and enhanced subtraction imaging for non-invasive and quantitative biological imaging. Nat. Nanotechnol., 15, 482. 10.1038/s41565-020-0678-5 32451501 PMC7307456

[B147] Weber J. S. Yang J. C. Atkins M. B. Disis M. L. (2015). Toxicities of immunotherapy for the practitioner. Jco 33, 2092–2099. 10.1200/JCO.2014.60.0379 PMC4881375 25918278

[B148] Weissleder R. (1999). Molecular imaging: exploring the next Frontier. Radiology 212, 609–614. 10.1148/radiology.212.3.r99se18609 10478223

[B149] Weissleder R. Ntziachristos V. (2003). Shedding light onto live molecular targets. Nat. Med. 9, 123–128. 10.1038/nm0103-123 12514725

[B150] Whiteside T. L. (2008). “Immune effector cells in the tumor microenvironment: their role in regulation of tumor progression,” in Innate and adaptive immunity in the tumor microenvironment (Dordrecht, The Netherlands: Springer), 1–33.

[B151] Wolfbeis O. S. (2015). An overview of nanoparticles commonly used in fluorescent bioimaging. Chem. Soc. Rev. 44, 4743–4768. 10.1039/c4cs00392f 25620543

[B152] Wu T.-L. Zhou D. (2011). Viral delivery for gene therapy against cell movement in cancer. Adv. Drug Deliv. Rev. 63, 671–677. 10.1016/j.addr.2011.05.005 21616108

[B153] Xiang J. Xu L. Gong H. Zhu W. Wang C. Xu J. (2015). Antigen-loaded upconversion nanoparticles for dendritic cell stimulation, tracking, and vaccination in dendritic cell-based immunotherapy. ACS Nano 9, 6401–6411. 10.1021/acsnano.5b02014 26028363

[B154] Xue H. Y. Liu S. Wong H. L. (2014). Nanotoxicity: a key obstacle to clinical translation of siRNA-based nanomedicine. Nanomedicine 9, 295–312. 10.2217/nnm.13.204 24552562 PMC4095781

[B155] Yang J. Liu H. Zhang X. (2014). Design, preparation and application of nucleic acid delivery carriers. Biotechnol. Adv. 32, 804–817. 10.1016/j.biotechadv.2013.11.004 24239630

[B156] Yang Y. Wang L. Cao H. Li Q. Li Y. Han M. (2019). Photodynamic therapy with liposomes encapsulating photosensitizers with aggregation-induced emission. Nano Lett. 19, 1821–1826. 10.1021/acs.nanolett.8b04875 30768274

[B157] Yatim N. Cullen S. Albert M. L. (2017). Dying cells actively regulate adaptive immune responses. Nat. Rev. Immunol. 17, 262–275. 10.1038/nri.2017.9 28287107

[B158] Yavuz C. T. Mayo J. T. Yu W. W. Prakash A. Falkner J. C. Yean S. (2006). Low-field magnetic separation of monodisperse Fe3O4 nanocrystals. Science 314, 964–967. 10.1126/science.1131475 17095696

[B159] Yoo D. Lee J.-H. Shin T.-H. Cheon J. (2011). Theranostic magnetic nanoparticles. Acc. Chem. Res. 44, 863–874. 10.1021/ar200085c 21823593

[B160] Yu G.-T. Rao L. Wu H. Yang L.-L. Bu L.-L. Deng W.-W. (2018). Myeloid-derived suppressor cell membrane-coated magnetic nanoparticles for cancer theranostics by inducing macrophage polarization and synergizing immunogenic cell death. Adv. Funct. Mater. 28, 1801389. 10.1002/adfm.201801389

[B161] Yu M. Duan X. Cai Y. Zhang F. Jiang S. Han S. (2019). Multifunctional nanoregulator reshapes immune microenvironment and enhances immune memory for tumor immunotherapy. Adv. Sci. 6, 1900037. 10.1002/advs.201900037 PMC6702652 31453054

[B162] Yuan Y. Gu Z. Yao C. Luo D. Yang D. (2019). Nucleic acid-based functional nanomaterials as advanced cancer therapeutics. Small 15, 1900172. 10.1002/smll.201900172 30972963

[B163] Zhan C. Huang Y. Lin G. Huang S. Zeng F. Wu S. (2019). A gold nanocage/cluster hybrid structure for whole-body multispectral optoacoustic tomography imaging, EGFR inhibitor delivery, and photothermal therapy. Small 15, 1900309. 10.1002/smll.201900309 31245925

[B164] Zhang C. Bu W. Ni D. Zhang S. Li Q. Yao Z. (2016). Synthesis of iron nanometallic glasses and their application in cancer therapy by a localized fenton reaction. Angew. Chem. Int. Ed. 55, 2101–2106. 10.1002/anie.201510031 26836344

[B165] Zhang F. Li F. Lu G.-H. Nie W. Zhang L. Lv Y. (2019). Engineering magnetosomes for ferroptosis/immunomodulation synergism in cancer. ACS Nano 13, 5662–5673. 10.1021/acsnano.9b00892 31046234

[B166] Zhang P. Hou Y. Zeng J. Li Y. Wang Z. Zhu R. (2019a). Coordinatively unsaturated Fe 3+ based activatable probes for enhanced MRI and therapy of tumors. Angew. Chem. Int. Ed. 58, 11088–11096. 10.1002/anie.201904880 31131511

[B167] Zhang P. Meng J. Li Y. Wang Z. Hou Y. (2019b). pH-sensitive ratiometric fluorescent probe for evaluation of tumor treatments. Materials 12, 1632. 10.3390/ma12101632 PMC6566363 31109039

[B168] Zhang X. Chen F. Turker M. Z. Ma K. Zanzonico P. Gallazzi F. (2020). Targeted melanoma radiotherapy using ultrasmall 177Lu-labeled α-melanocyte stimulating hormone-functionalized core-shell silica nanoparticles. Biomaterials 241, 119858. 10.1016/j.biomaterials.2020.119858 32120314 PMC7171978

[B169] Zhang Y. Xiao J. Sun Y. Wang L. Dong X. Ren J. (2018). Flexible nanohybrid microelectrode based on carbon fiber wrapped by gold nanoparticles decorated nitrogen doped carbon nanotube arrays: in situ electrochemical detection in live cancer cells. Biosens. Bioelectron. 100, 453–461. 10.1016/j.bios.2017.09.038 28963962

[B170] Zhao H. Xu J. Li Y. Guan X. Han X. Xu Y. (2019). Nanoscale coordination polymer based nanovaccine for tumor immunotherapy. ACS Nano 13, 13127–13135. 10.1021/acsnano.9b05974 31710460

[B171] Zhao P. Tang Z. Chen X. He Z. He X. Zhang M. (2019). Ferrous-cysteine-phosphotungstate nanoagent with neutral pH fenton reaction activity for enhanced cancer chemodynamic therapy. Mater. Horiz. 6, 369–374. 10.1039/c8mh01176a

[B172] Zheng D.-W. Lei Q. Zhu J.-Y. Fan J.-X. Li C.-X. Li C. (2017). Switching apoptosis to ferroptosis: metal-organic network for high-efficiency anticancer therapy. Nano Lett. 17, 284–291. 10.1021/acs.nanolett.6b04060 28027643

[B173] Zheng L. Hu X. Wu H. Mo L. Xie S. Li J. (2020). *In vivo* monocyte/macrophage-hitchhiked intratumoral accumulation of nanomedicines for enhanced tumor therapy. J. Am. Chem. Soc. 142, 382–391. 10.1021/jacs.9b11046 31801020

[B174] Zhou Z. Liu X. Zhu D. Wang Y. Zhang Z. Zhou X. (2017). Nonviral cancer gene therapy: delivery cascade and vector nanoproperty integration. Adv. Drug Deliv. Rev. 115, 115–154. 10.1016/j.addr.2017.07.021 28778715

[B175] Zhuo H. Zheng B. Liu J. Huang Y. Wang H. Zheng D. (2018). Efficient targeted tumor imaging and secreted endostatin gene delivery by anti-CD105 immunoliposomes. J. Exp. Clin. Cancer Res. 37, 42. 10.1186/s13046-018-0712-8 29499713 PMC5833054

